# Emerging harmful algal blooms caused by distinct seasonal assemblages of a toxic diatom

**DOI:** 10.1002/lno.12189

**Published:** 2022-10-07

**Authors:** Alexa R. Sterling, Riley D. Kirk, Matthew J. Bertin, Tatiana A. Rynearson, David G. Borkman, Marissa C. Caponi, Jessica Carney, Katherine A. Hubbard, Meagan A. King, Lucie Maranda, Emily J. McDermith, Nina R. Santos, Jacob P. Strock, Erin M. Tully, Samantha B. Vaverka, Patrick D. Wilson, Bethany D. Jenkins

**Affiliations:** ^1^ Department of Cell and Molecular Biology University of Rhode Island Kingston Rhode Island; ^2^ Department of Biomedical and Pharmaceutical Sciences College of Pharmacy, University of Rhode Island Kingston Rhode Island; ^3^ Graduate School of Oceanography University of Rhode Island Narragansett Rhode Island; ^4^ Rhode Island Department of Environmental Management Office of Water Resources Providence Rhode Island; ^5^ Fish and Wildlife Research Institute Florida Fish and Wildlife Conservation Commission St. Petersburg Florida; ^6^ Woods Hole Center for Oceans and Human Health Woods Hole Oceanographic Institution Woods Hole Massachusetts; ^7^ College of Earth, Ocean and Atmospheric Sciences Oregon State University Corvallis Oregon

## Abstract

Diatoms in the *Pseudo‐nitzschia* genus produce the neurotoxin domoic acid. Domoic acid bioaccumulates in shellfish, causing illness in humans and marine animals upon ingestion. In 2017, high domoic acid levels in shellfish meat closed shellfish harvest in Narragansett Bay, Rhode Island for the first and only time in history, although abundant *Pseudo‐nitzschia* have been observed for over 60 years*.* To investigate whether an environmental factor altered endemic *Pseudo‐nitzschia* physiology or new domoic acid‐producing strain(s) were introduced to Narragansett Bay, we conducted weekly sampling from 2017 to 2019 and compared closure samples. Plankton‐associated domoic acid was quantified by LC‐MS/MS and *Pseudo‐nitzschia* spp. were identified using a taxonomically improved high‐throughput rDNA sequencing approach. Comparison with environmental data revealed a detailed understanding of domoic acid dynamics and seasonal multi‐species assemblages. Plankton‐associated domoic acid was low throughout 2017–2019, but recurred in fall and early summer maxima. Fall domoic acid maxima contained known toxic species as well as a novel *Pseudo‐nitzschia* genotype. Summer domoic acid maxima included fewer species but also known toxin producers*.* Most 2017 closure samples contained the particularly concerning toxic species, *P. australis*, which also appeared infrequently during 2017–2019. Recurring *Pseudo‐nitzschia* assemblages were driven by seasonal temperature changes, and plankton‐associated domoic acid correlated with low dissolved inorganic nitrogen. Thus, the Narragansett Bay closures were likely caused by both resident assemblages that become toxic depending on nutrient status as well as the episodic introductions of toxic species from oceanographic and climatic shifts.

Phytoplankton photosynthesis sustains oceanic food webs and generates nearly half of photosynthetically fixed carbon on Earth (Falkowski 1994). Despite their key roles in ocean ecosystems, some phytoplankton generate toxins that are harmful to animal and human health, including diatoms in the genus *Pseudo‐nitzschia*. *Pseudo‐nitzschia* have caused harmful algal blooms (HABs) in locations across the globe (Hasle 2002; reviewed by Bates et al. 2018; reviewed by Anderson et al. 2019). *Pseudo‐nitzschia* spp. produce domoic acid, a water‐soluble, neuroexcitatory toxin that serves as an agonist of glutamate receptors in the central nervous system of animals (Bates et al. 1989). Domoic acid disrupts marine food webs as it bioaccumulates in vectors such as copepods, anchovies, and shellfish, causing mortality in sea birds and marine mammals (Work et al. 1993; Lefebvre et al. 1999; Tammilehto et al. 2015). Domoic acid ingestion in humans causes amnesic shellfish poisoning (ASP) with symptoms such as seizures and permanent short‐term memory loss. Deaths from ASP resulted from the first known domoic acid event in 1987 on Prince Edward Island, Canada (Bates et al. 1989). However, there may be hidden ASP fatalities and cases due to unconsidered differential diagnosis (reviewed by Bates et al. 2018). Long‐term exposure to low domoic acid concentrations via high shellfish diets may also be harmful, causing diminished memory (Grattan et al. 2018). In addition to human health impacts, domoic acid events have far‐reaching effects on coastal communities including public panic and massive income losses in shellfish sales (Hoagland et al. 2002).

Although domoic acid shellfish closures have frequently occurred along the Gulf of Mexico and the Pacific coasts of the United States and Canada, they have not occurred in northeastern US waters until very recently (reviewed by Bates et al. 2018). As summarized by Bates et al. (2018), the first northeastern US closure due to domoic acid exceeding regulatory action limits in shellfish meat was in the Gulf of Maine in September 2016 which resulted in the market recall of shellfish product. This 2016 closure in Maine was followed by precautionary closures in Massachusetts and Rhode Island (RI) in October 2016 (Fig. S1A). Less than a year later in March 2017, a second northeastern closure occurred, which was limited to only Narragansett Bay, RI with domoic acid levels in shellfish meat exceeding the 20 *μ*g domoic acid g^−1^ regulatory limit (Fig. S1B). Subsequent closures followed in fall 2017 through winter 2018 in eastern and western Maine (reviewed by Bates et al. 2018), and again in the fall of 2018 and 2019 (Clark et al. 2021). Overall, domoic acid closures due to toxic *Pseudo‐nitzschia* spp. are an emergent problem in the northeastern US.

Narragansett Bay is a unique platform for gaining insights into causative drivers of *Pseudo‐nitzschia* HABs. It is the location of one of the longest running plankton time series in the world, the Narragansett Bay Long‐Term Plankton Time Series (NBPTS; Fig. 1) (https://web.uri.edu/gso/research/plankton/; Smayda and The Bunker C Community 1959–1997). The NBPTS data show *Pseudo‐nitzschia* spp. have been present for over 60 years and often at high cell abundances (Fig. 2). Species within the *Pseudo‐nitzschia* genus differ in their ability to produce toxin, making species identification important for HAB monitoring (reviewed by Bates et al. 2018). However, little is known about the *Pseudo‐nitzschia* species composition in Narragansett Bay. *Pseudo‐nitzschia* spp. are enumerated by the NBPTS using light microscopy which is insufficient for species delineation as many are cryptic (e.g., Orsini et al. 2004; Lundholm et al. 2006; Quijano‐Scheggia et al. 2009). Therefore, it is difficult to assess the presence or absence of toxigenic species in historical Narragansett Bay data. Transmission electron microscopy has been used on selected samples from Narragansett Bay and nearby waters and has identified six toxigenic species that can co‐occur: *P. delicatissima*, *P. fraudulenta*, *P. multiseries*, *P. pseudodelicatissima*, *P. pungens* var. *pungens*, and the typically offshore *P. seriata* (Hargraves and Maranda 2002). Given that domoic acid closures have occurred only recently despite the persistence of *Pseudo‐nitzschia* spp. and domoic acid monitoring since the early 1990s, it is critical to understand the ecological species dynamics and domoic acid production of *Pseudo‐nitzschia* spp. in Narragansett Bay.

**Fig. 1:**
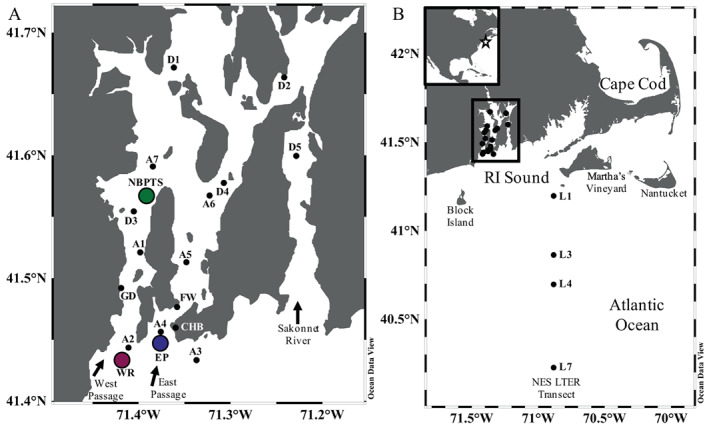
Sites within Narragansett Bay, RI, USA and offshore Northeast U.S. Shelf Long‐Term Ecological Research (NES LTER) cruise stations sampled from 2016 to 2019. (**A**) Sample locations (*n* = 18) within Narragansett Bay including the Long‐Term Plankton Time Series (NBPTS) at the green point, University of RI Graduate School of Oceanography dock (GD), Whale Rock (WR) at the purple point, East Passage (EP) as the blue point, Castle Hill beach (CHB), and Fort Wetherill (FW), and sites sampled by RI harmful algal bloom (HAB) monitoring in March 2017 (D1–D5) and sites sampled with vertical net tows (A1–A7). (**B**) Inset shows overview of sampling site locations in North America. The location of the within Narragansett Bay sites are outlined by the black box. Select stations were sampled along the routine NES LTER transect (L1, L3, L4, L7). The figure was made in Ocean Data View (Schlitzer 2002).

**Fig. 2:**
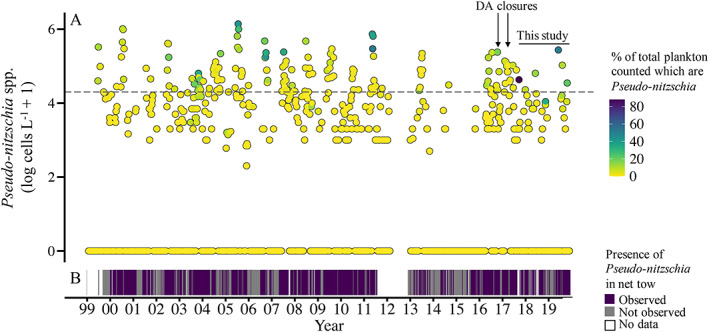
Twenty‐year record of the presence of *Pseudo‐nitzschia* spp. in Long‐Term Plankton Time Series (NBPTS) samples in Narragansett Bay, RI (**A**) Live *Pseudo‐nitzschia* spp. cell abundance observed at the NBPTS site from approximately weekly sampling from January 1999 to November 2019 (*n* = 1003). Values are log_10_‐transformed, with +1 cell to all values to create non‐zero values in the transformation. Surface water samples were used in 2000, 2001, and 2008–2019, and mixed surface and depth (~ 7 m) samples were used in 1999 and 2002–2007. No sampling occurred from March 2012 to December 2012. The horizontal dashed line is the log‐transformed RI HAB action threshold of 20,000 cells L^−1^. The line annotated “this study” is the timeframe in which samples were taken for DNA sequencing and particulate domoic acid analysis. Arrows indicate the 2016 precautionary closure and 2017 closure due to domoic acid (DA), respectively. The color of the points indicates the percentage of total plankton community counts that *Pseudo‐nitzschia* spp. represent. (**B**) Data from 20 *μ*m vertical net tow samples (~ 7 m bottom depth) corresponding to count data presented in a; each line represents whether *Pseudo‐nitzschia* spp. were observed or not (*n* = 920). Net tow data unavailable from September 2011 to March 2012.

Several hypotheses can explain the recent domoic acid closures in Narragansett Bay. It is possible that a particularly toxigenic species of *Pseudo‐nitzschia* was recently introduced or became much more prevalent. Alternatively, or in addition, environmental dynamics may have shifted to increase toxin production by resident species. Toxigenic species regulate domoic acid production in response to a variety of factors including silicate limitation (Bates et al. 1996), phosphate limitation (Pan et al. 1996), and different nitrogen sources (Auro and Cochlan 2013). Domoic acid production may also be regulated in response to multifactor combinations such as silicate limitation and high pCO_2_ levels (Tatters et al. 2012). Thus, one or more environmental factors may have stimulated domoic acid production in Narragansett Bay.

To delineate the relationships between the environment, *Pseudo‐nitzschia* species composition, and domoic acid production, we conducted weekly time series sampling at several sites in Narragansett Bay from 2017 to 2019 (Fig. 1). Offshore sampling transects were used to examine linkages between Narragansett Bay species assemblages and those on the Northeast US Shelf (NES). Particulate domoic acid from plankton samples (> 5 *μ*m size) was directly quantified with tandem mass spectrometry coupled with liquid chromatography (LC‐MS/MS). Typically, in RI HAB monitoring, LC‐MS/MS is not employed in high frequency sampling but instead used as confirmation for other measurements, such as ELISA‐based methods or the Scotia Rapid Test. These frequent and highly sensitive particulate domoic acid measurements from LC‐MS/MS provided an unprecedented understanding of toxin dynamics, especially in terms of understanding the ecology of toxin distribution at levels below closure concerns and deciphering toxin congeners.

*Pseudo‐nitzschia* species composition was delineated using new molecular barcoding methodology targeting the 18S–5.8S rDNA internal transcribed spacer region 1 (ITS1). This high‐throughput sequencing approach allowed for species to be cataloged at high temporal resolution, and circumvented labor intensive and time‐consuming electron microscopy. In addition, we built upon previously designed approaches that employ ITS1 amplification with fragment size polymorphism to distinguish *Pseudo‐nitzschia* species (Hubbard et al. 2008, 2014). We targeted the ITS1 region with a custom primer designed from an updated database of global *Pseudo‐nitzschia* rDNA sequences to yield unique sequences from at least 41 different species. Overall, comparing particulate domoic acid production and species composition in toxic, nontoxic, and 2016 and 2017 shellfish harvest closure samples along with environmental variables informed our ecological understanding of *Pseudo‐nitzschia* HABs in Narragansett Bay. We have begun to predict their temporal patterns and magnitude from recurring patterns in this time series, which has potential implications for the larger region of the Atlantic Northeast. Ultimately, our results will inform forecasting HAB models and management decisions of RI shellfish harvest.

## Materials and methods

### Sample sites

Narragansett Bay is a temperate estuary in RI, USA with seasonally reoccurring phytoplankton blooms in the winter and smaller blooms in the summer (Oviatt 2004). The Providence River contributes inflow of freshwater and urban nutrients in the north, and Atlantic Ocean water is introduced from RI Sound in the south into the East and West Passage mouths of Narragansett Bay. From September 2017 to November 2019 (referred to as the “study period” in figures and text), surface seawater samples were collected weekly or biweekly at multiple sites in Narragansett Bay (Fig. 1; Table S1; Schlitzer 2002). Weekly sampling was conducted at the mid‐Bay NBPTS site (41.57°N, 71.39°W). The Whale Rock site at the mouth of West Passage (41.34°N, 71.42°W) is the location of the URI GSO Narragansett Bay Fish Trawl Survey and samples were collected at this location starting in October 2018. The East Passage site (41.45°N, 71.38°W) is at the East Passage mouth and a location for RI HAB monitoring. During spring and fall time periods when *Pseudo‐nitzschia* spp. cells were observed or particulate domoic acid was detected, additional sampling was conducted on the same day at the NBPTS and Whale Rock sites along with East Passage. Otherwise, the frequency of each sample was about once a week. Additional sampling was conducted at shore‐based sites: Castle Hill Beach (East Passage shore access), Fort Wetherill (East Passage shore access), and GSO Dock (NBPTS alternative site) (Table S1). Vertical net tow samples from various sites in Narragansett Bay were also collected by L. Maranda in fall 2017, summer–fall 2018, and spring–fall 2019, with corresponding particulate domoic acid Scotia Rapid Test analysis and *Pseudo‐nitzschia* spp. cell counts, from an average of 1.7 m^3^ water concentrated and zooplankton removed by filtering across a 150 *μ*m mesh. These samples are referred to as “net tow samples” in text. Surface seawater samples from the 2017 closure were collected by RI DEM on 13 March 2017 from five sites in mid‐Bay and sent to K. Hubbard for DNA extraction; however, all these sites were north of the March 2017 shellfish harvest closure area. In addition, four NES LTER transect cruises onboard the R/V *Endeavor* provided offshore surface samples in 2018 and 2019 (Table S1).

### Environmental metadata

Surface seawater temperature and salinity were measured using YSI Sondes (YSI/Xylem): EXO Sonde from May to August 2018 at sites outside the NBPTS and Whale Rock, ProDSS starting September 2018, and 6920 V2 for weekly NBPTS and Fish Trawl Survey samples from October 2018 to September 2019. Data from the NBPTS are available: https://web.uri.edu/gso/research/plankton/data/, as are 2017 Fish Trawl Survey data: https://web.uri.edu/gso/research/fish-trawl/data/. Fish Trawl Survey data from 2018–2019 at Whale Rock were acquired from the fish trawl personnel. Chlorophyll *a* (Chl *a*) measurements were determined by vacuum filtering triplicate surface seawater onto GF/F filters (0.6–0.8 *μ*m particle retention; Whatman n.k.a. Cytiva). Filters were extracted in 90% acetone for 24 h in the dark at −20°C. After 24 h, samples were equilibrated at room temperature for 20 min, vortexed, and decanted for fluorometric reading in a 10 AU fluorometer (Turner Designs) for May–July 2018 and NBPTS samples or a Trilogy fluorometer (Turner Designs) for samples August 2018–October 2019. Chl *a* was determined as described in the Turner Designs manual by subtracting values from samples acidified with three drops of 10% hydrochloric acid (Turner 2019).

Samples for dissolved nutrient analysis were filtered through 0.2‐*μ*m polyethersulfone filters (Sterlitech) and frozen at −20°C until analysis one to 22 months later on a Lachat QuickChem 8500 (Hach) at the URI Marine Science Research Facility (Narragansett, RI) or on AA3 (SEAL Analytical) at the University of Washington Nutrient Analysis Facility (Seattle, WA) including duplicate samples for comparison between facilities. The UW AA3 measures nitrate directly, whereas the Lachat nitrate values were calculated from nitrite + nitrate − nitrite. Any negative Lachat values for ammonium were changed to zero, but all other values were used in analysis. For any analysis showing DIN, the summation of ammonium, nitrate, and nitrite was used.

**Fig. 3:**
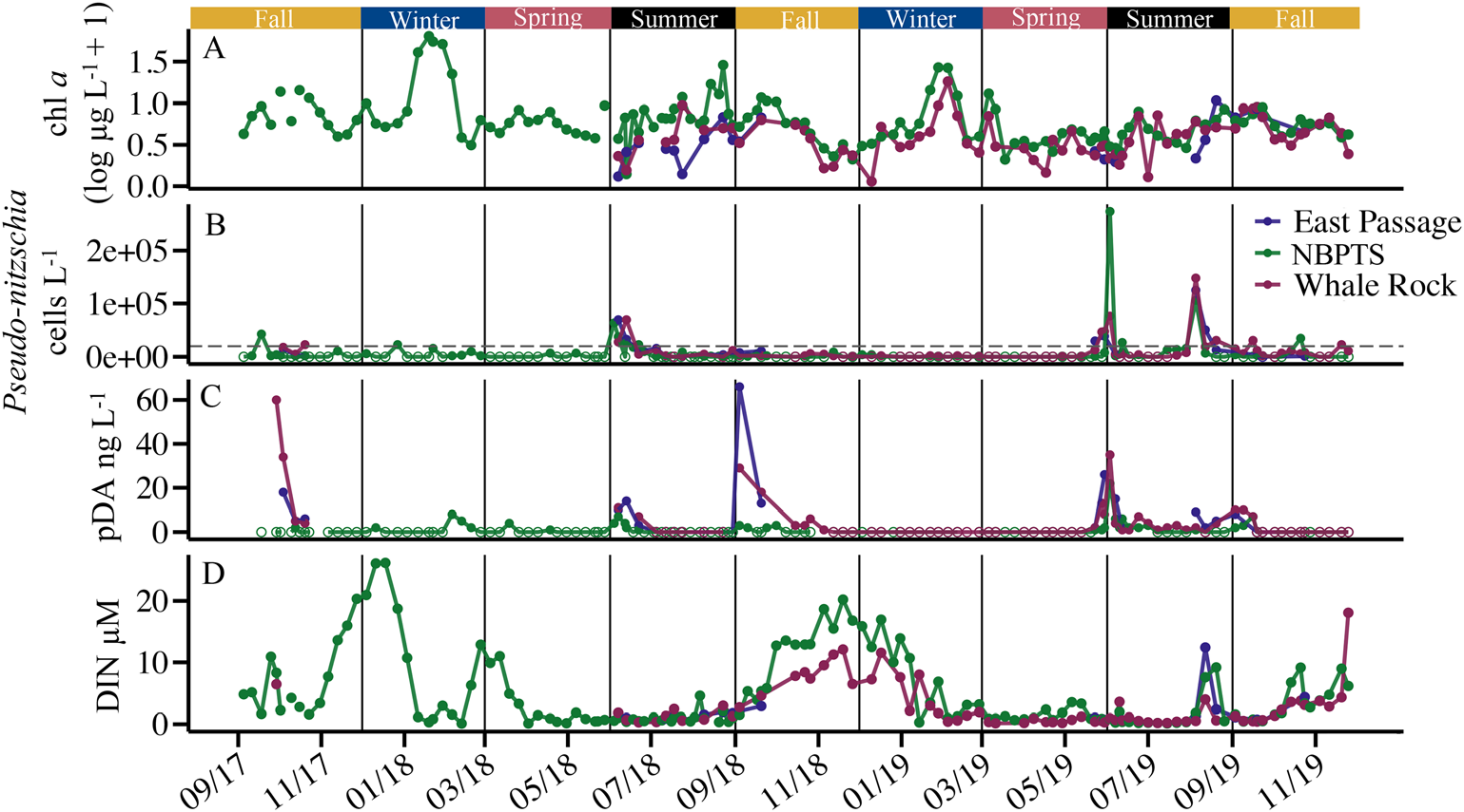
Comparison of total chlorophyll *a* (Chl *a*) concentration, *Pseudo‐nitzschia* spp. cell counts, particulate domoic acid (pDA), and dissolved inorganic nitrogen (DIN) at the most frequently sampled sites during the study period. The East Passage site was usually sampled in the summer and fall only. For details on species assemblages associated with domoic acid maxima and at the Long‐Term Plankton Time Series (NBPTS) site, please *see* Figs. 6, S6, respectively. (**A**) Trends of Chl *a* across time at the East Passage site (*n* = 22), the NBPTS site (*n* = 131), and Whale Rock site (*n* = 69). Values are log_10_‐transformed with + 1 added to all values to create only positive values in the transformation. (**B**) *Pseudo‐nitzschia* spp. cell counts across time at East Passage (*n* = 26), NBPTS (*n* = 141), and Whale Rock (*n* = 73). The dashed line is the 20,000 cells L^−1^ action threshold for RI HAB monitoring. Open circles denote the absence of cells. (**C**) Patterns of pDA measured across time at East Passage (*n* = 26), NBPTS (*n* = 131), and Whale Rock (*n* = 72). Open circles denote no pDA was detected. (**D**) DIN, which is the summation of nitrite, nitrate, and ammonium measured, across time at East Passage (*n* = 23), NBPTS (*n* = 136), and Whale Rock (*n* = 71).

Between 15 and 50 mL of whole seawater were preserved with final concentration of 1% acidic Lugol's solution in glass or plastic containers (with a silica bead [5‐mm rattler plating bead, Zymo Research] later added to prevent degradation of diatoms stored in plastic). Samples were stored at 4°C for less than a year before being counted. One milliliter of sample was placed in a Sedgewick‐Rafter counting chamber (Science First/Wildco), *Pseudo‐nitzschia* spp. cells were identified at the genus level and counted using 20X phase contrast light microscopy on a BX40 microscope (Olympus America). *Pseudo‐nitzschia* spp. cell counts from the NBPTS were conducted on live samples using the same enumeration protocol on an Eclipse E800 (Nikon Instruments). Cell counts conducted on the same‐day as NBPTS sampling were preferentially used over counts from archived samples from September 2017 to November 2019 for the NBPTS site in Figs. 2, 3 except for any samples corresponding to additional excursions by our team at that site. The % of total plankton counted which are *Pseudo‐nitzschia* in Fig. 2 were determined by dividing the number of *Pseudo‐nitzschia* spp. cells counted by the total plankton abundance reported for the sample and multiplying that by 100. *Pseudo‐nitzschia* were recorded as observed or not observed in net tow samples (> 20 *μ*m). Current and historical NBPTS cell abundance data are available online: https://web.uri.edu/gso/research/plankton/. The action threshold of *Pseudo‐nitzschia* spp. cell counts of 20,000 cells L^−1^ is highlighted in some figures, which is from the RI HAB Plan (2020).

### *Pseudo‐nitzschia* spp. ITS1 DNA analysis

Plankton biomass for *Pseudo‐nitzschia* spp. DNA identification was collected with a peristaltic pump, passing an average of 270 mL of surface seawater across a 25‐mm 5.0‐*μ*m polyester membrane filter (Sterlitech). Widths of some *Pseudo‐nitzschia* spp. are < 5.0 *μ*m (reviewed in Lelong et al. 2012), but the 5‐*μ*m size pore likely captured chains and horizontally orientated cells and was consistent with pore size used to measure particulate domoic acid. Filters were flash frozen in liquid nitrogen and stored at −80°C until DNA extraction. DNA was extracted using a modified version of the DNeasy Plant DNA Extraction Kit (Qiagen) with an added bead beating step for 1 min and QIA‐Shredder column (Qiagen) as reported in Chappell et al. (2019). In addition, DNA was eluted into 30 *μ*L of elution buffer with a second elution step of either 30 or 15 *μ*L of elution buffer to maximize DNA yield. DNA was assessed for quality with a Nanodrop spectrophotometer (Thermo Fisher Scientific) and quantified using a Qubit fluorometer (Invitrogen) with the Broad Range dsDNA and High Sensitivity dsDNA Kits (Thermo Fisher Scientific). DNA yields ranged from below the limit of detection to 26.5 ng *μ*L^−1^, with an average of 2.0 ng DNA *μ*L^−1^ eluent. NBPTS samples (October 2016 and March 2017) had an average of 300 mL surface seawater passed through a 25‐mm 0.2‐*μ*m filter. These were extracted following existing NBPTS methods of DNA extraction using the DNeasy Blood and Tissue Kit (Qiagen) with an added bead beating step (Canesi and Rynearson 2016) which yielded an average of 0.9 ng DNA *μ*L^−1^ eluent as measured by the Qubit. Net tow samples had 50 mL of concentrate passed across a 0.22‐*μ*m pore size Sterivex filter unit (MilliporeSigma), and were extracted with the same modified DNeasy Plant DNA extraction protocol as above, with four times volumes of AP1 buffer and RNase A and beads added to the unit to account for the larger sample surface area, extraction occurring within the capped unit itself to maximize yield, and then the lysate removed with a sterile syringe and subsequent steps with adjusted volumes as appropriate. As expected, DNA yields were higher from the Sterivex units ranging from 2.4 to 54.0 ng DNA *μ*L^−1^ eluent with an average of 13.7 ng DNA *μ*L^−1^ per elution. For the 13 March 2017 Narragansett Bay samples, 125 mL of surface seawater was passed across a HV filter and DNA was extracted with the Qiagen DNeasy Plant Mini Kit, with an additional step of using scissors to shred each filter in the lysis buffer (Hubbard et al. 2008). Scissors were sterilized with DNA AWAY (Thermo Fisher Scientific), followed by 70% ethanol and sterile water, and then autoclaved before each use. As measured by the Qubit, the average DNA yield was 3.7 ng DNA *μ*L^−1^ per elution.

The 18S V4 rDNA region was initially amplified using primers specific to diatoms (Zimmermann et al. 2011); however, this highly conserved V4 region was not able to fully delineate *Pseudo‐nitzschia* species, but the ITS1 region discriminates among *Pseudo‐nitzschia* spp. (Hubbard et al. 2008). To target the *Pseudo‐nitzschia* ITS1 we used an existing eukaryotic ITS1 forward primer: 5′‐TCCGTAGGTGAACCTGCGG‐3′ (White et al. 1990) and designed a custom reverse primer (Pn‐ITS1R) against a conserved 5.8S region using 132 *Pseudo‐nitzschia* ITS1 sequences (Table S2) (NCBI nucleotide database as of 3 April 2019): 5′‐CATCCACCGCTGAAAGTTGTAA‐3′. We added MiSeq adapters to the 5′ ends of these primers and the full primers sequences were: forward: 5′‐TCGTCGGCAGCGTCAGATGTGTATAAGAGACAGTCCGTAGGTGAACCTGCGG‐3′ and reverse: 5′‐GTCTCGTGGGCTCGGAGATGTGTATAAGAGACAGCATCCACCGCTGAAAGTTGTAA‐3′.

*Pseudo‐nitzschia* species expected to amplify with these primers are summarized (Table S2). The expected ranges for PCR products were from 235–370 bp as the size of the ITS1 region differs for some *Pseudo‐nitzschia* taxa. PCR amplification details are provided in the Supporting Information S1 (*see* “*Pseudo‐nitzschia* spp. ITS PCR methods”). Sequence library preparation and 2 × 250 bp Illumina MiSeq (Illumina) sequencing were performed by the RI Genomics and Sequencing Center (Kington, RI). There were 193 environmental samples sequenced, along with the negative and positive controls (*see* Supplemental Information [Controls for sequencing]), for a total of 196 samples using two sets of MiSeq indices on the same sequencing plate. The raw sequencing reads are available on NCBI's Short Read Archive (Bioproject PRJNA690940).

Sequences were analyzed with a custom bioinformatics pipeline. Illumina MiSeq adapters and primers were trimmed from both read ends using CutAdapt v1.15 (Martin 2011) and input to DADA2 v1.16 (30) to determine amplicon sequence variants (ASVs). Reads lacking ITS1 primer sequences were discarded. Unique ASVs, some differing by 1 bp, were included in subsequent analysis*. Pseudo*‐*nitzschia* sequence identification utilized a manually curated database from NCBI sequences (Table S2), which included sequences from species such as *P. caciantha*, *P. calliantha*, *P. cuspidata*, *P. delicatissima*, *P. galaxiae*, and *P. pseudodelicatissima* cross referenced with transmission and scanning electron microscopy of those species in culture (Lundholm et al. 2003; Amato et al. 2007). While not every sequence included in the curated database was verified from electron microscopy, we assessed database sequence similarity through a MAFFT alignment in Geneious Prime v2021.0.1 (Biomatters, Ltd.) as another way to assess sequence identity and similarity independently from the reported NCBI taxonomic assignments (Katoh et al. 2002; Katoh and Standley 2013). ASVs were identified as *Pseudo‐nitzschia* taxa using the scikit‐learn naïve Bayes machine learning classifier (Pedregosa et al. 2011) at default settings in QIIME2 v2020.2 (Bolyen et al. 2019) which included a confidence level of 0.7. The scikit‐learn naïve Bayes machine learning classifier identified 94 ASVs from environmental samples as *Pseudo‐nitzschia* at the species level. Additional *Pseudo‐nitzschia* ASVs were assigned using a BLAST pipeline; details are provided in Supporting Information S1 (*see* “Taxonomic determination of *Pseudo‐nitzschia* ASVs using BLAST”). All ASVs identified in this study as *Pseudo‐nitzschia* were deposited into NCBI GenBank under accession numbers MW447658‐MW447770.

ASV counts were transformed into relative abundance out of total ASVs assigned to *Pseudo‐nitzschia*. If an ASV accounted for < 1% relative abundance in a sample, it was then considered “not present” to avoid potentially spurious results. This step removed 60 ASVs from consideration. The remaining 53 ASVs were analyzed in a presence/absence matrix to avoid potential problems from inflating read numbers with cell counts and rDNA copy number variation (Jenkins and Bertin 2021*a*).

### Domoic acid analysis

At each sampling station, biomass was collected to match the plankton size fraction used for DNA sequencing and maximize collection volume by filtering approximately 2 L of surface seawater across a 47‐mm 5.0‐*μ*m polyester membrane filter (Sterlitech). Filters were flash frozen in liquid nitrogen and stored at −80°C until extraction. Filters were extracted for 4 h in 0.1 M acetic acid, vigorously vortexing each hour. A series of spike and recovery experiments with the domoic acid standard and filters consistently showed > 95% domoic acid recovery. This extraction method also allowed for dual analysis of domoic acid via Scotia tests and LC‐MS/MS. Extracts were passed through a 0.2‐*μ*m syringe filter directly into a 1.5 mL LC‐MS vial for LC‐MS/MS analysis on a Prominence UFLC system (Shimadzu) coupled to a SCIEX 4500 QTRAP mass spectrometer (AB Sciex). Mass spectrometry methodological details are provided in the Supporting Information S1. Plankton‐associated domoic acid was quantified to ng particulate domoic acid L^−1^ of filtered seawater using an external calibration curve performed from pure domoic acid standards of increasing concentrations (Sigma‐Aldrich), included in each analysis. Domoic acid data and environmental metadata are publicly available through the NSF Biology and Chemical Oceanography Data Management Office, BCO‐DMO (Jenkins and Bertin 2021*b*) and visualized across time at each site sampled in Fig. S2.

Domoic acid was also analyzed in the tissue of six live mussels (RI DEM Scientific Collector's Permit #435) collected at the GSO dock on 5 June 2019 during a period of increased *Pseudo‐nitzschia* cell abundance and elevated particulate domoic acid (*see* Supporting Information S1 for methodological details). Scotia rapid tests were performed on the offshore A3 site net tow samples using approximately 950 mL of the net tow concentrate.

### Statistical analysis and visualization

For the principal component analyses (PCA), any sample from a site with particulate domoic acid measurements was used, including samples not sequenced. Variables were tested for independence by analyzing autocorrelation between variables with *r*‐squared values of scatterplots between the variables. The only variables that were potentially autocorrelated were DSi and DIP (*R* ^2^ = 0.68), but because we could not ascertain whether their source to Narragansett Bay was similar (e.g., river vs. point source inputs), they were kept separate. Data were transformed on a log scale using the addition of a constant = 1, except for surface water temperature where the constant 10 was used and for salinity where no constant was needed. Each variable was standardized by subtracting the mean from its value and then dividing by the standard deviation (in factoextra, scale = TRUE) (Kassambara and Mundt 2020). Levene's test was performed using the car package v3.0.10 in R with command leveneTest() (Fox and Weisberg 2019), one‐way ANOVA was run in base R with aov(), and Tukey HSD was performed with TukeyHD() in base R. The function BIOENV (Best Subset of Environmental Variables) through the vegan bioenv() command was used to determine the best model formula from the scaled environmental variables used in the PCA for 127 sequenced Narragansett Bay samples of the Bray‐Curtis distance of the *Pseudo‐nitzschia* ASVs both by season and throughout the study period (Clarke and Ainsworth 1993).

Similarities between the presence/absence of Jaccard distance of *Pseudo‐nitzschia* ASVs across samples were explored with analysis of similarities (ANOSIM) and nonmetric multidimensional scaling (NMDS) (Jaccard 1912). A test for dispersion of groups was performed prior to ANOSIM, to assess reliability of ANOSIM results. Dispersion of groups was tested using command betadisper() in the R package vegan. Then, within the same package, permutest() performed on those results with 999 permutations and alpha value of 0.05 was used to assess significance. Only groups lacking a significant group dispersion were carried forward into ANOSIM. Groups investigated included season, sample group, month, year, sample site, closure time, particulate domoic acid grouping, and if *Pseudo‐nitzschia* spp. cells were over or under the action threshold. For seasonal groupings, the Northern Hemisphere meteorological groupings were used which starts a season on the first of the month in which the respective equinox or solstice begins. Thus, seasons were designated as summer (June, July, and August), fall (September, October, and November), winter (December, January, and February), and spring (March, April, and May). Since many groups had uneven numbers, ANOSIM was preferred to PERMANOVA tests. ANOSIM was run using anosim() in R package vegan, with 999 permutations and an alpha value of 0.05. NMDS plots of the Jaccard distance of the presence or absence of the *Pseudo‐nitzschia* ASVs which passed through the > 1% relative abundance per sample threshold were made to examine similarities and dissimilarities in species composition of samples across sampling sites, seasons, and particulate domoic acid concentrations. NMDS solutions were not reached by defaults, so number of iterations was increased (trymax = 100) and number of dimensions was raised to 3 (*k* = 3), but still visualized in 2D. The final stress of the NMDS was 0.0924. Data was visualized in R v4.0.2 (R Core Team 2020) in R Studio v1.1.456 (RStudio 2016) with packages: vegan v2.5.6 (Dixon 2003) for group dispersion tests and ANOSIM; ggplot2 v3.3.2 (Wickham 2016) for plots such as line graphs and heatmap; phyloseq v1.32.0 (McMurdie and Holmes 2013) for ASV data set manipulation like subsetting and transformations, and NMDS analysis; factoextra v1.0.7 (Kassambara and Mundt 2020) for PCA of data related to particulate domoic acid; and viridis v0.5.1 for continuous scale coloring (Garnier 2018). Colors were chosen to maximize color blind accessibility (Tol 2018).

## Results

In response to the domoic acid‐triggered 2016 precautionary closure and 2017 closure, we initiated a time series study of particulate domoic acid and *Pseudo‐nitzschia* spp. composition from September 2017 to November 2019, resulting in over 230 samples collected at various sites within Narragansett Bay and offshore RI Sound and the Atlantic Ocean (Fig. 1). The majority of samples were collected at the NBPTS site in mid‐Narragansett Bay, Whale Rock at the West Passage mouth, and East Passage at its corresponding mouth of Narragansett Bay (Fig. 1A). At seven additional sites ranging from mid‐Narragansett Bay to offshore, vertical plankton net tows (20 *μ*m) were conducted to examine and compare depth‐integrated *Pseudo‐nitzschia* spp. assemblages (Fig. 1A). To understand how offshore dynamics may be impacting *Pseudo‐nitzschia* spp. and particulate domoic acid dynamics in Narragansett Bay, we sampled four sites from four NES long‐term ecological research (LTER) research cruises (Fig. 1B). *Pseudo‐nitzschia* spp. composition was also assessed in archived closure samples: five samples each from the 2016 precautionary closure and the 2017 closure at the NBPTS site and five samples collected by the RI HAB monitoring program in March 2017 at sites in mid to upper Narragansett Bay (Fig. 1A). No LC‐MS/MS particulate domoic acid measurements were made for these closure samples, as samples were not available. For most samples, light microscopy counts of *Pseudo‐nitzschia* spp. cells at the genus level were recorded, extracted Chl *a* was used to evaluate total phytoplankton biomass, and filtered seawater was analyzed for inorganic nutrient concentrations.

### Historical and contemporary *Pseudo‐nitzschia* spp. abundance in Narragansett Bay

From 1999 to 2019, *Pseudo‐nitzschia* spp. abundance at the NBPTS site ranged from unobserved to 1,389,000 cells L^−1^ (Fig. 2A). Cell abundances exceeding the RI HAB monitoring program action threshold of 20,000 cells L^−1^ (RI HAB Plan 2020) occurred annually except in 2015 and comprised approximately 16% of counted samples. Elevated *Pseudo‐nitzschia* spp. abundance was observed in summer and fall, and *Pseudo‐nitzschia* cells made up over 50% of the total enumerated plankton community in May 2011, September 2017, and June 2019 (Fig. 2A). In addition to these weekly direct plankton counts in 1 mL of seawater, the presence and absence of species were recorded from higher volume net tows. While *Pseudo‐nitzschia* spp. typically represented a low fraction of the total plankton cells enumerated in 1 mL surface seawater (averaging 1%) and were detected in less than half of 1 mL counted samples, *Pseudo‐nitzschia* spp. were consistently detected in over 68% of the high volume NBPTS net tow samples (Fig. 2B), thus indicating *Pseudo‐nitzschia* spp. were frequently present in Narragansett Bay, but at concentrations less than 1000 cells L^−1^.

*Pseudo‐nitzschia* spp. abundance was uncoupled from maxima in phytoplankton biomass as measured by Chl *a* concentration, demonstrating that *Pseudo‐nitzschia* spp. bloom at distinct times from other phytoplankton (Fig. 3A,B). During our time series study period from September 2017 to November 2019, *Pseudo‐nitzschia* spp. abundance and seasonal patterns were similar across the NBPTS site and the East and West entrances of Narragansett Bay (Fig. 3B). Peak *Pseudo‐nitzschia* spp. cell abundance occurred in the summers, and abundance was elevated in two of the 3 years of fall samples (Fig. 3B). Cell counts exceeded the action threshold annually, with the exception of the East Passage site in 2017 when only three samples were collected (Fig. 3B). Abundance ranged from unobserved to 148,000 cells L^−1^ at Whale Rock and to 125,000 cells L^−1^ at East Passage (Fig. 3B).

### Seasonal patterns of particulate domoic acid

Our time series data revealed extended periods of low or undetectable particulate domoic acid concentrations in Narragansett Bay with distinct seasonal maxima in May, June, September and/or October of each year (Fig. 3C, S3). During the late spring and early summer of 2018 and 2019, particulate domoic acid concentrations were similar at all three sites and corresponded to elevated *Pseudo‐nitzschia* spp. cell counts (Fig. 3B,C). In contrast, in the fall, elevated particulate domoic acid occurred with low *Pseudo‐nitzschia* spp. abundance (Fig. 3B,C). In addition, in the fall of 2017 and 2018, particulate domoic acid maxima occurred at both the East (East Passage) and West (Whale Rock) Narragansett Bay entrances, but not at the mid‐Bay NBPTS site (Fig. 3C). The Narragansett Bay entrances also had the highest particulate domoic acid concentrations measured during our time series with 60 ng particulate domoic acid L^−1^ at Whale Rock in October 2017 and 66 ng particulate domoic acid L^−1^ at East Passage in September 2018 when *Pseudo‐nitzschia* spp. cell counts were below the action threshold (Fig. 3B,C). Although the particulate domoic acid measured during our study period did not occur at levels triggering concomitant shellfish harvest closures, domoic acid was detected in the tissues of all six mussels sampled on 5 June 2019, during a period of increased *Pseudo‐nitzschia* spp. cell numbers and elevated particulate domoic acid concentrations. Toxin ranged from 0.4–4 ng domoic acid g^−1^ shellfish meat, and the highest concentration of 4 ng domoic acid g^−1^ was well below the mandatory closure level of 20 *μ*g domoic acid g^−1^ shellfish meat (NSSP 2019).

### Toxin and low dissolved nitrogen concentrations

**Fig. 4:**
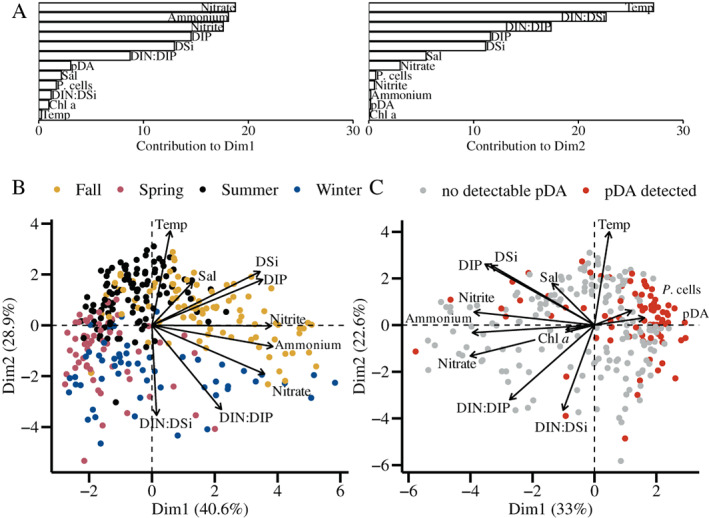
Principal component analysis (PCA) biplot of all surface seawater samples from Narragansett Bay, RI during the study period from any sampling site, excluding NES LTER stations, with full portfolio of physical and chemical parameters log‐transformed and standardized. Parameters include surface seawater salinity (Sal), surface seawater temperature (temp), chlorophyll *a* concentration (Chl *a*), dissolved inorganic silicate (DSi), dissolved inorganic phosphorus (DIP), nitrate, nitrite, ammonium, and nutrient ratios related to the summation of those nitrogen sources as dissolved inorganic nitrogen (DIN) of DIN : Si and DIN : P, *Pseudo‐nitzschia* spp. cell abundance (*P*. cells), and particulate domoic acid (pDA) concentration. (**A**) Bar chart of the contribution of each variable to dimension 1 and dimension 2 corresponding to the PCA shown in **C**. (**B**) Samples colored by the season sampled, from September 2017 to November 2019, excluding Chl *a*, *P*. cells, and pDA (*n* = 312). (**C**) Samples colored by whether pDA was detected (red) or not detected (gray) by LC‐MS/MS, and including Chl *a*, *P*. cells, and pDA (*n* = 238).

A principle component analysis (PCA) was used to correlate the chemical and biological properties of all samples collected during this study (Fig. 4). Data were log‐transformed and standardized and included particulate domoic acid concentrations, *Pseudo‐nitzschia* spp. cell counts, Chl *a* concentrations, dissolved nutrient concentrations (inorganic nitrogen [DIN]: ammonium, nitrite, nitrate, inorganic phosphorus [DIP], and inorganic silicate [DSi]), surface seawater salinity and temperature. Nitrate, ammonium, and nitrite contributed most to the first component, while temperature, DIN : DSi, and DIN : DIP primarily contributed to the second component (Fig. 4A). Temperature, as expected, positively correlated with summer and fall samples and negatively correlated with winter and spring samples (Fig. 4B). *Pseudo‐nitzschia* spp. cell counts and particulate domoic acid concentrations contributed little to variability in the whole dataset and were correlated with each other (Fig. 4). *Pseudo‐nitzschia* spp. cell counts and particulate domoic acid concentrations showed an inverse relationship to Chl *a* concentrations, nitrate, and ammonium (Fig. 4C). Nitrate and ammonium were negatively correlated with a large cluster of spring, summer, and fall samples in which particulate domoic acid was detected (Fig. 4C). Notably, increased particulate domoic acid concentrations were typically synchronous with low DIN concentrations throughout the time series (cf. Fig. 3C,D) and 82% of samples with detectable particulate domoic acid occurred when DIN was less than 5 *μ*M (Fig. S4).

### *Pseudo‐nitzschia* spp. delineated with ITS1 rDNA ASVs

The ITS region is one of the most distinctive genetic identifiers for *Pseudo‐nitzschia* spp., revealing intraspecific diversity which has implications for toxin production (Casteleyn et al. 2008; Hubbard et al. 2008; Kaczmarska et al. 2008). We used high‐throughput sequencing and ASVs of the ITS1 reads to identify species and some strains as ASVs can distinguish differences as little as one base pair (Callahan et al. 2016). Sequencing yielded an average of approximately 35,550 reads with 131 ASVs per sample. In total, there were 6503 ASVs recovered across the 192 samples excluding sequencing controls. The ITS1 reverse primer was designed to maximize the number of *Pseudo‐nitzschia* spp. amplified, and as such was also expected to amplify other genera. Of the top 100 ASVs recovered, 20 were from *Pseudo‐nitzschia* ranging from the top 2 to the top 70 ASVs. Sequences from diatoms outside the *Pseudo‐nitzschia* genus and other plankton such as dinoflagellates were also recovered in the top 100 most numerous ASVs. Thirty of the top 100 most numerous ASVs had no significant similarity to existing sequences in the National Center for Biotechnology Information (NCBI) nucleotide database and may represent unknown diversity within the marine plankton, or alternatively species lacking ITS1 data.

ASVs were identified as *Pseudo‐nitzschia* taxa using a curated database from NCBI sequences (Table S2) and the scikit‐learn naïve Bayes machine learning classifier (Pedregosa et al. 2011) which identified 97 ASVs as *Pseudo‐nitzschia* at the species level. The number of reads belonging to *Pseudo‐nitzschia* spp. in each sample ranged from 19 to 46,314 with an average of 10,655. Nineteen known *Pseudo‐nitzschia* species were represented by 53 ASVs. Individual *Pseudo‐nitzschia* species ranged from having one to seven ASVs (Table 1). Most taxa represented by two or more ASVs had a dominant ASV found in over double the number of samples compared to the other ASVs of that taxon. However, three taxa had two ASVs which were equally dominant throughout samples: *P. americana*, *P. calliantha*, and *P. subpacifica*. One group of three similar ASVs were only identified at the genus level for this analysis, and likely represented potential novel diversity related to *P. americana* from 95% similar identity via megablast results.

**Table 1:** Summary of the 53 *Pseudo‐nitzschia* spp. amplicon sequence variants (ASVs) recovered from Narragansett Bay 2016–2019 samples, including offshore Northeast Shelf Long‐Term Ecological Research (NES LTER) cruise samples (*n* = 192), that are present at 1% or higher relative abundance in individual samples out of all *Pseudo‐nitzschia* ASVs. Detection was determined by the presence of ASVs in this study compared to previous geographic reports summarized in Bates et al. (2018).

Taxa	% present in samples	# of ASVs	Observed domoic acid producer, (reviewed in Bates et al. 2018)	First detection along U.S. Atlantic Coast, (reviewed in Bates et al. 2018)	Cell width (*μ*m)
*P. multiseries*	92	2	Yes	No	3.5–4.8*
*P. calliantha*	91	7	Yes	No	1.1–2.6*
*P. americana*	80	4	No	No	2.5–4.5*
*P. pungens* var. *pungens*	73	2	Yes	No	2.2–5.4*
*P. plurisecta*	39	2	Yes	No	1.5–2.0†
*P. delicatissima*	31	3	Yes	No	1.0–2.4*
*P*. sp. Group 1‡	29	3	‐	‐	‐
*P. fraudulenta*	27	2	Yes	No	4.0–8.0*
*P. australis*	23	2	Yes	No	4.6–10.0*
*P. subpacifica*	20	3	Yes	No	5.0–7.0*
*P. pungens* var. *aveirensis*	16	1	No	Yes	‐
*P. galaxiae*	11	6	Yes	Yes	1.0–1.8*
*P. hasleana*	10	1	Yes	Yes	1.5–2.8*
*P. cuspidata*	5	3	Yes	No	1.0–2.0*
*P. turgidula*	4	1	Yes	No	2.5–5.0*
*P. inflatula*	3	2	No	Yes	1.3–2.0*
*P. caciantha*	2	3	Yes	Yes	2.7–3.5*
*P. heimii*	2	2	No	No	3.5–6.0*
*P. multistriata*	2	2	Yes	Yes	2.5–3.7*
*P. lineola*	1	1	No	Yes	2.0–2.7*
*P. fryxelliana*	1	1	No	Yes	2.1–2.5*

^†^ Reported in Orive et al. (2013).

^‡^ Group of three ASVs most similar to *P. americana* sequences with an average of 95% identity.

Because of high variability in ITS1 rDNA copy number and unknown copy number per *Pseudo‐nitzschia* strain, this study could not equate or transform ITS1 reads to absolute abundance, or even relative abundance, of *Pseudo‐nitzschia* species. For example, quantitative PCR has been used before to determine ITS1 copy number in eight *Pseudo‐nitzschia* isolates and a significant variation of two orders of magnitude was found between 16 copies in a *P. delicatissima* isolate to 748 in *P. multiseries* (Hubbard et al. 2014). Thus, in our analyses we used the presence or absence of individual *Pseudo‐nitzschia* ITS1 ASVs to circumvent the copy number challenge and examine co‐occurring taxa.

### Seasonal cycle of *Pseudo‐nitzschia* spp. diversity

**Fig. 5:**
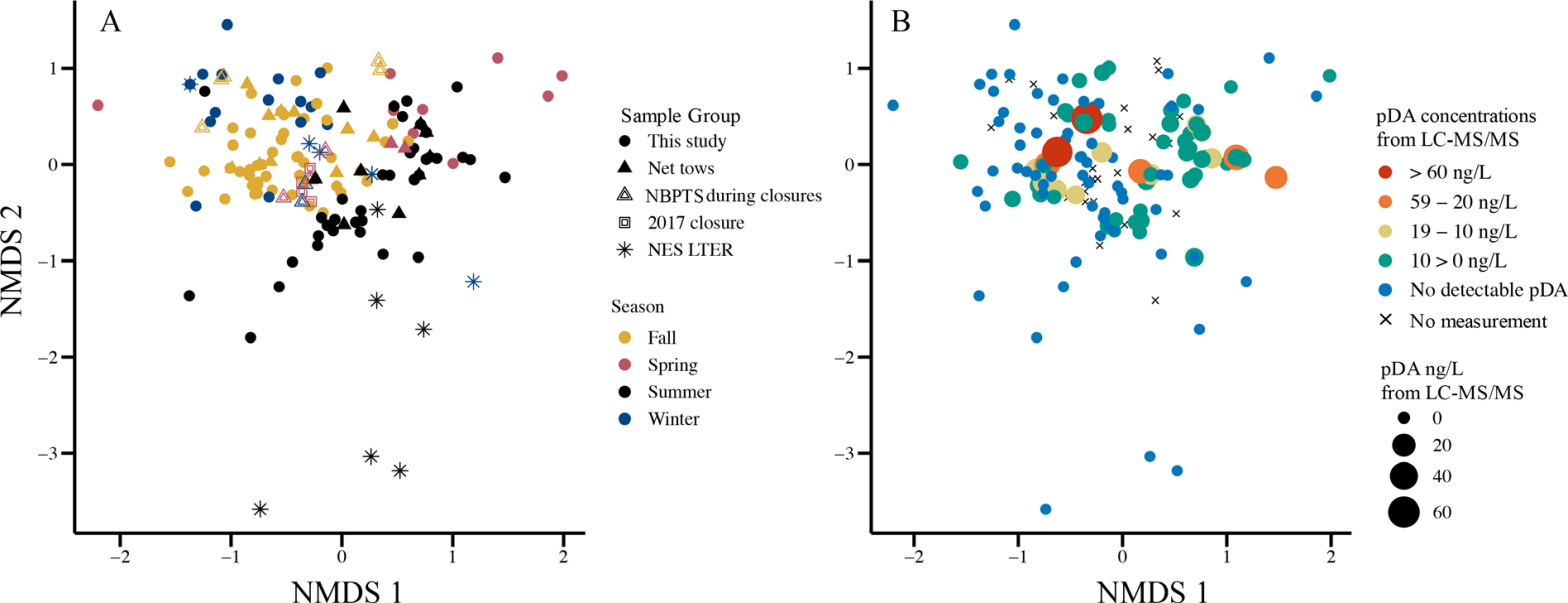
Nonmetric multidimensional scaling (NMDS) of Jaccard distance matrix of the presence or absence of *Pseudo‐nitzschia* spp. ASVs in samples from Narragansett Bay, RI and offshore (*n* = 192). Both plots are the same NMDS with stress = 0.0924. Solution was not reached by defaults, so iterations raised to 100 and dimensions were raised to three but visualized in two dimensions. (**A**) *Pseudo‐nitzschia* spp. assemblages across seasons (color) by sample group (shape), including the Narragansett Bay Long‐Term Plankton Time Series (NBPTS) and Northeast Shelf Long‐Term Ecological Research (NES LTER) offshore cruises, which has different sample types and sample years. (**B**) Assemblages of *Pseudo‐nitzschia* taxa overlaid with concentration of particulate domoic acid (pDA) measured in filtered seawater L^−1^ from LC‐MS/MS measurements. Samples where no measurements were taken for LC‐MS/MS are indicated with an x, including net tow, precautionary closure, closure, and some NES LTER samples.

*Pseudo‐nitzschia* spp. composition was compared in all samples. Season was the only reliable grouping of ASVs to test for ANOSIM as determined by dispersion of group tests (*n* = 192, *p* = 0.771). ANOSIM showed a significant difference between *Pseudo‐nitzschia* assemblages during different seasons (*p* = 0.001, Fig. 5A). NMDS of the Jaccard distance matrix of the presence and absence of *Pseudo‐nitzschia* ASVs showed assemblages were similar across most sites sampled in the same season, including the offshore NES LTER samples (Fig. 5A). In addition, there was a group of summer NES LTER samples which did not group with other Narragansett Bay samples (Fig. 5A). Adjacent seasons shared taxa as shown by overlapping points, and there was a general pattern across an annual trajectory connecting adjacent seasons (Fig. 5A). Exceptions to this general seasonal trend were spring closure samples from 2017 that grouped with fall samples from 2018 and 2019 (Fig. 5A).

**Fig. 6:**
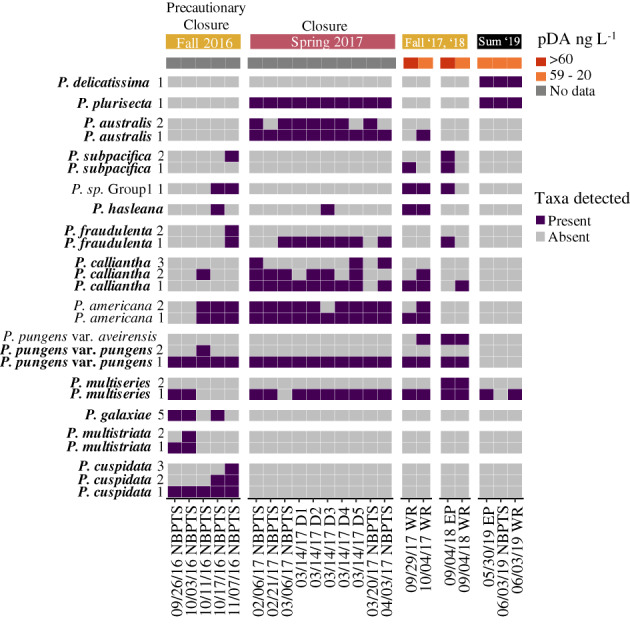
*Pseudo‐nitzschia* spp. detected in samples around the 2016 precautionary closure, the 2017 closure, and from the seven most toxic samples during the September 2017 to November 2019 study period in Narragansett Bay, RI. Corresponding particulate domoic acid (pDA) concentrations from September 2017 to November 2019 are shown in the top row. The 2016 precautionary closure (7 October 2016–29 October 2016) and 2017 closure (26 February 2017–24 March 2017) samples included those collected at the Narragansett Bay Long‐Term Plankton Time Series (NBPTS) site and RI DEM HAB monitoring sites (D1–D5). Samples from the study period shown include those collected at NBPTS, Whale Rock (WR), and East Passage (EP) during fall 2017, fall 2018, and summer (sum) of 2019. Distinct ASVs identified as the same species were kept separate and denoted with numbers. A group of ASVs in the data set which could only be identified to the genus level, are labeled as “*P*. sp. Group1.” Purple shading indicates ASVs which occurred at > 1% relative abundance per sample as present and gray as those not present at < 1% relative abundance or absent in an individual sample. Species known to produce domoic acid according to Bates et al. (2018) are denoted in bold. Particulate domoic acid values (ng L^−1^ seawater filtered) were 60 (WR—29 September 2017), 34 (WR—4 October 2017), 66 (EP—4 September 2018), 29 (WR—30 May 2019), 26 (EP—30 May 2019), 22 (NBPTS—3 June 2019), and 35 (WR—3 June 2019).

The *Pseudo‐nitzschia* assemblages co‐occurring with elevated particulate domoic acid concentrations (> 10 ng particulate domoic acid L^−1^) in the spring, summer, and fall were distinct from each other (Fig. 5). However, there was some overlap of taxa, such as *P. multiseries*, throughout seasons (Figs. 5, 6). Interestingly, within a season, similar assemblages both do and do not produce domoic acid (Fig. 5). From the BIOENV analysis of the environmental variables of temperature, salinity, DSi, DIP, ammonium, nitrate, nitrite, DIN:DIP, and DIN:Si, it was determined that temperature, DIP, nitrate, and DIN:DIP best correlated with the whole *Pseudo‐nitzschia* ASV dataset (*n* = 127 samples, correlation = 0.44). When considering only the fall samples, temperature and DIN : DIP best correlated with the fall *Pseudo‐nitzschia* ASVs (*n* = 41, correlation = 0.57), winter samples correlated with salinity, DIN:DIP, and DIN:Si (*n* = 16, correlation = 0.63), spring samples correlated with temperature, DIP, nitrite, DIN:Si (*n* = 18, correlation = 0.69), and summer samples correlated with salinity, temperature, DIP, and Si (*n* = 52, correlation = 0.44).

*Pseudo‐nitzschia* species richness followed a seasonal cycle (Fig. S5). From Levene's test, it was determined that the number of ASVs by seasonal groupings displayed homogeneity of variances (*p* > 0.05), following the assumption appropriate for a one‐way ANOVA. There was a significant difference in the number of ASVs recovered from samples by season as determined by a one‐way ANOVA (*p* < 0.001), and it was determined from Tukey multiple pair‐wise comparisons that fall compared to the other seasons had significant differences in the number of ASVs (*p* < 0.01). Samples with the highest number of ASVs occurred from August to October, and diversity peaked again in March and decreased in December–January and May (Fig. S5). The number of *Pseudo‐nitzschia* ASVs in Narragansett Bay samples ranged from 1 to 13 with an average of six ASVs present. The average number of ASVs recovered from the 2016 precautionary closure and 2017 closure samples was higher at eight ASVs. There was no difference between diversity measures when cell counts were under or over the action threshold or when particulate domoic acid was detected or not.

### Multi‐species assemblages of *Pseudo‐nitzschia* spp.

Fourteen known domoic acid‐producing taxa were found in Narragansett Bay (Table 1). Some of these toxigenic *Pseudo‐nitzschia* spp. (*P. multiseries*, *P. calliantha*, and *P. pungens* var. *pungens*) were frequent and found in 70% or more of the total 2017–2019 Narragansett Bay samples (Table 1). The non‐toxigenic *P. americana* was also found in approximately 70% or more of the total 2017–2019 Narragansett Bay samples. The 2016 precautionary closure samples had a lower diversity of toxic *Pseudo‐nitzschia* (*P. pungens* var. *pungens* and *P. cuspidata)* species compared to the higher diversity found in all 2017 closure samples (*P. pungens* var. *pungens*, *P. multiseries*, *P. americana*, *P. calliantha*, *P. fraudulenta*, *P. plurisecta*, and *P. australis*) (Fig. 6). In this study, ASVs were recovered belonging to eight taxa previously unreported along the U.S. Atlantic coast (Table 1). Four taxa (*P. pungens* var. *aveirensis*, *P. galaxiae*, *P. multistrata*, and *P. fryxelliana*) had exact matches between ASVs and database sequences, one species (*P. hasleana*) had 99% similarity, two species (*P. inflatula* and *P. caciantha*) has 98% similarity, and *P. lineola* had 85% similarity. Of these newly documented species, *P. hasleana*, *P. galaxiae*, and *P. multistriata* were present in the closure samples from 2016 (Fig. 6) and from 2017 to 2019, *P. pungens* var. *aveirensis* was present in the fall and co‐occurred with *P. pungens* var. *pungens* (Fig. S6). The other newly observed taxa were rarely observed (Fig. S6).

The presence of rare, but highly toxic *Pseudo‐nitzschia* spp. may augment particulate domoic acid in Narragansett Bay. Specifically, *P. australis* is one of the most toxic *Pseudo‐nitzschia* species (reviewed by Trainer et al. 2012), and it was present in every 2017 closure sample (Fig. 6) and the NES LTER samples with detected particulate domoic acid (Fig. S7). In contrast, *P. australis* was absent in the 2016 precautionary closure and infrequent in the 2017–2019 Narragansett Bay samples and the other NES LTER samples (Figs. 6, S6–S8). The 2016 precautionary closure also contained ASVs from *P. multistriata*, *P. cuspidata*, and *P. galaxiae* that were rarely found in the 2017–2019 Narragansett Bay samples (Figs. 6, S6).

## Discussion

### Patterns of particulate domoic acid repeat seasonally under closure thresholds

In Narragansett Bay, *Pseudo‐nitzschia* spp. producing domoic acid are a newly emergent HAB problem. In this study, a combination of high frequency sampling at several locations, sensitive quantitative particulate domoic acid measurements and new species delineation methods advanced our understanding of the ecological dynamics of *Pseudo‐nitzschia* spp. and toxin production in Narragansett Bay. The rapid deployment of LC‐MS/MS measurements allowed for near real‐time detection of particulate domoic acid as well as the ability to adaptively sample additional sites during occurrences of increasing particulate domoic acid. The LC‐MS/MS method is over two orders of magnitude more sensitive (LOQ = 1 ng particulate domoic acid L^−1^ seawater filtered) than the Scotia Rapid Tests employed by RI HAB monitoring for the detection of domoic acid in the plankton (LOD = 300 ng particulate domoic acid L^−1^ filtered seawater; Jellet et al. 2006). Thus, the repeating seasonal maxima of particulate domoic acid revealed in this study would not have been detected by routine RI DEM HAB monitoring. The advantage of this LC‐MS/MS method for uncovering HAB dynamics was illustrated during spring 2019. From 12 November 2018 to 20 May 2019, particulate domoic acid was consistently undetectable at all sites (Fig. 3C). Beginning 20 May 2019, low particulate domoic acid was detected at the mouth of Narragansett Bay. Sampling frequency was increased, and the following week (28 May 2019), particulate domoic acid levels were 10‐fold higher at the Narragansett Bay entrances (Fig. 3C). There was continued detection of particulate domoic acid throughout June 2019, and we captured the entire event of particulate domoic acid increasing, reaching its maximum, and decreasing back to undetectable levels (Fig. 3C).

Our time series sampling from 2017 to 2019 showed maxima in particulate domoic acid that increased repeatedly in the fall and summer, generally for 2–3 weeks (Fig. 3C). These data, along with the 2016 fall precautionary closure, indicate the times of the year when domoic acid production was elevated and potentially a problem in Narragansett Bay. These patterns would have been missed by solely relying on enumeration of *Pseudo‐nitzschia* spp. cells alone, which were much lower than RI DEM action thresholds during the 2017–2019 time series (cf. Fig. 3B,C). The frequent and highly sensitive particulate domoic acid measurements allowed us to better understand toxin dynamics that would have been masked by using elevated cell counts as a proxy. The highest particulate domoic acid concentration (66 ng L^−1^) measured in our study was similar to particulate domoic acid levels in the Gulf of Maine in 2012 (60 ng L^−1^), but much lower than in the 2016 Gulf of Maine *P. australis* bloom (37.5 *μ*g L^−1^) (Clark et al. 2019). Even though the particulate domoic acid detected during our study period was below closure concerns, our data show that domoic acid is seasonally present, recurs, and could be a future concern.

### Season and temperature structure *Pseudo‐nitzschia* spp. assemblages

When we initiated our study in 2017, we hypothesized that the domoic acid closures in Narragansett Bay were either caused by the entrainment of a particularly toxic species typically not present at high abundances in Narragansett Bay or by changing environmental conditions augmenting toxin production in resident *Pseudo‐nitzschia* spp. assemblages. We have demonstrated support for both hypotheses. The new ITS1 high‐throughput sequencing methods deployed in this study both uncovered new *Pseudo‐nitzschia* species diversity and identified strains of the same species. We identified species not previously known in Narragansett Bay, as well as eight species previously unreported along the U.S. Atlantic coast (Table 1). New *Pseudo‐nitzschia* spp. diversity uncovered by using high‐throughput sequencing is similar to findings from another Narragansett Bay molecular study that demonstrated newly observed species of the diatom genus *Thalassiosira* (Rynearson et al. 2020). The application of high‐throughput sequencing with the ITS1 builds on the utility of the ARISA approach as *Pseudo‐nitzschia* species with the same ITS fragment lengths (e.g., *P. australis* and *P. seriata*) can be discriminated as well as additional species identified (e.g., *P. calliantha* and *P. hasleana*) (Hubbard et al. 2008, 2014).

Several co‐occurring species were found frequently in more than 70% of samples from 2017–2019. We considered these species common residents of Narragansett Bay: *P. multiseries*, *P. pungens* var. *pungens*, and *P. calliantha* (Table 1). Notably, these three species were also present during the 2016 precautionary closure (Fig. 6). Our study also showed that recurring resident *Pseudo‐nitzschia* assemblages in Narragansett Bay can be driven to toxin production. Distinct multi‐species assemblages of *Pseudo‐nitzschia* grouped by season and recurred interannually (Fig. 5). Assemblages sampled within a season had different particulate domoic acid concentrations despite containing similar species (Fig. 5).

*Pseudo‐nitzschia* spp. have been observed to persist across the annual range of temperatures experienced in Narragansett Bay (Miller and Kamykou 1986; Dortch et al. 1997; Delegrange et al. 2018). During the 2017–2019 sampling period, Narragansett Bay surface water temperature ranged from −1.1°C to 25.5°C, and *Pseudo‐nitzschia* spp. were observed year‐round. Low temperatures may be controlling the mostly nontoxic winter assemblage, which included *P. americana* (Figs. 4C, S6). However, particulate domoic acid in Narragansett Bay was detected across temperatures ranging from 3.1°C to 24°C. Temperature is likely a major driving force of the seasonal species groupings, and has been shown to influence other diatom assemblages in Narragansett Bay (Karentz and Smayda 1984; Canesi and Rynearson 2016; Rynearson et al. 2020). It is notable that the water temperatures were markedly different during the 2016 and 2017 closures with 15–20°C in October 2016 and 1–5°C in March 2017 (https://web.uri.edu/gso/research/plankton/) and each contained different species assemblages (Figs. 5, 6). These differences demonstrate that species assemblages present under different Narragansett Bay temperatures matter more for domoic acid production potential than does temperature itself as a direct driver of domoic acid production.

### Nitrogen availability may vary particulate domoic acid production by similar *Pseudo‐nitzschia* spp. assemblages

The repeating summer and fall particulate domoic acid maxima correlated with low DIN concentrations (Figs. 3, 4, S4). Therefore, nutrient dynamics may enhance toxin production in toxin‐capable assemblages in Narragansett Bay. There have been other observations of increased domoic acid production under nitrogen‐limiting conditions, depending on species, cell cycle stage, and nitrogen substrate (reviewed by Trainer et al. 2012). For example, strains of *P. multiseries* produce domoic acid under nitrogen‐limiting conditions with a variety of nitrogen substrates (Hagström et al. 2011; reviewed by Trainer et al. 2012). In Narragansett Bay, *P. multiseries* was a common member of both the toxic summer and fall assemblages that occurred during periods of low DIN (Figs. 3D, S6). It seems counter‐intuitive to consider that nitrogen stress would lead to elevated domoic acid production in *Pseudo‐nitzschia* spp. as nitrogen is a requirement for the production of domoic acid as glutamate, which results from nitrogen assimilation, is a precursor for domoic acid biosynthesis in *P. multiseries* (Brunson et al. 2018). However, studies on the remodeling of nitrogen metabolism under nitrogen stress in the non‐domoic acid producing diatom *Phaeodactylum tricornutum* have shown that enzymes in glutamine biosynthesis are upregulated by nitrogen limitation (Levitan et al. 2015). In addition, a switch from DIN to urea as a nitrogen source may support *Pseudo‐nitzschia* growth and increased toxicity as well (Howard et al. 2007; Kudela et al. 2008). Currently, it is unknown how urea, an anthropogenic nitrogen source, impacts domoic acid production in Narragansett Bay, which may be useful to examine in future studies.

In addition to a relationship between low DIN and elevated particulate domoic acid, there may be multiple co‐occurring ecological factors eliciting species‐specific responses of cell growth and toxin production. Notably, strains of the same *Pseudo‐nitzschia* spp. may respond differently (Thessen et al. 2009; Sahraoui et al. 2011; Markina and Aizdaicher 2016). In Narragansett Bay, strain‐specific responses may play a key role in toxin production, as most species had more than two ASVs assigned (Table 1). For example, the fall species assemblages produced the highest particulate domoic acid during our 2017–2019 study period. This could be attributed to toxin production by unique species constituents distinct from the spring assemblages, or other conditions in addition to low DIN forcing greater toxin production in species common to both fall and spring such as *P. multiseries* or *P. pungens* var. *pungens* (Fig. S6). Complicating the challenge of deciphering ecological factors contributing to domoic acid production is the long‐standing mystery of why some *Pseudo‐nitzschia* spp. produce domoic acid and whether domoic acid production confers an evolutionarily or ecological benefit (reviewed in Zabaglo et al. 2016). Other biotic factors unexamined in this study could also influence toxin production such as bacterial communities (Bates et al. 1995; Sison‐Mangus et al. 2016) and grazing pressure (reviewed by Bates et al. 2018).

### *P. australis* as a species of concern

In Narragansett Bay, the absence of domoic acid closures prior to 2016–2017 was likely not due to a lack of monitoring or under sampling. RI HAB monitoring involves three levels: an action threshold for *Pseudo‐nitzschia* spp. cell counts, the Scotia Rapid Test for particulate domoic acid in the plankton, and a management threshold for domoic acid in shellfish meat. Sampling is expansive with 38 sites ranging from offshore Block Island, RI to coastal ponds to upper Narragansett Bay. This effort totals over 300 discrete samples annually, which were concentrated from 20 L of surface seawater across a 20 *μ*m plankton net (RI HAB Plan 2020).

In October 2016, the first HAB‐biotoxin shellfish harvest closure due to elevated plankton‐associated domoic acid lasted for 26 days in Narragansett Bay (Fig. S1A). It was a precautionary closure since domoic acid in the shellfish meat did not exceed the management threshold; however, domoic acid was detected in wild shellfish meat, including quahogs (*Mercenaria mercenaria*) and mussels (*Mytilus edulis*). During the 2016 closure period, nine toxigenic species were found at the NBPTS site, but notably, *P. australis* was absent (Fig. 6). Samples from nearby Massachusetts during the same time period on 11 October 2016 included multi‐species assemblages with *P. pungens* var. *pungens* and others, as well as an absence of *P. australis*. Surprisingly, this differs from *P. australis* present in Maine and Canada during their coinciding 2016 closures (reviewed by Bates et al. 2018; Clark et al. 2019).

In 2017, a second closure in Narragansett Bay occurred when domoic acid was detected in shellfish meats collected near the Narragansett Bay entrances adjacent to RI coastal waters, and *P. australis*, which may have originated from offshore populations, was present in all samples sequenced (Figs. 6, S1B). The presence of *P. australis*, with possible additional toxin contributions from *P. multiseries*, *P. plurisecta*, *P. fraudulenta*, *P. calliantha*, and *P. pungens* var. *pungens*, likely was responsible for the 2017 closure. From 2017 to 2019, *P. australis* was not commonly observed, except for fall 2017, winter and spring 2018, and fall 2019 when particulate domoic acid was less than 10 ng L^−1^ (Fig. S6). The winter 2018 samples coincided with offshore NES LTER sampling, where *P. australis* was present at all stations and the station closest to Narragansett Bay had 15 ng particulate domoic acid L^−1^ (Fig. S7A). Altogether, these data suggest that *P. australis* is not a resident species of Narragansett Bay, but instead likely introduced from offshore, which is consistent with its likely import from the Scotian Shelf into the Gulf of Maine in 2016 (Clark et al. 2021). This taxon may be particularly problematic in regard to domoic acid production in Narragansett Bay. Perhaps in 2016, the geographic barrier of Cape Cod or differing water masses separated *Pseudo‐nitzschia* spp. assemblages, up and down the Atlantic Northeast. Therefore, one Maine and Canada assemblage contained *P. australis* sourced from the Labrador current (Clark et al. 2021) and a different toxic assemblage was present in RI and Massachusetts. As the Grand Banks can be a boundary between the Gulf Stream and Labrador current (Gonçalves Neto et al. 2021), this could explain the distinct communities between Narragansett Bay and the Gulf of Maine. Similar to Clark et al. 2021, hydrodynamic modeling and lagrangian particle tracking could be employed to show the most likely source of *P. australis* to Narragansett Bay in 2017. The trend of an expanded geographic range for *P. australis* may be part of a larger pattern in the Atlantic Ocean: *P. australis* expanded into northern European waters (reviewed by Trainer et al. 2012) and was not documented until 1994 in northwest Spain (Míguez and Fernlindez 1996) or until 1999 in Scottish waters (Gallacher et al. 2001).

The likely introduction of *P. australis* into Narragansett Bay may have been driven by climate change along the Northwest Atlantic shelf: warmer and saltier water (Gonçalves Neto et al. 2021) augmented by the decreased nitrogen loading within Narragansett Bay. Along the US Pacific Coast, increased *P. australis* growth rates were linked to climate change‐related warm water anomalies and low nutrients from a stratified euphotic zone (McCabe et al. 2016; Ryan et al. 2017). *P. australis* persisted in the nutrient‐depleted stratified waters and achieved high growth rates when nutrients were resupplied from upwelling (McCabe et al. 2016; McKibben et al. 2017; Ryan et al. 2017). Notably, in Narragansett Bay, nitrogen loading declined significantly from recent implementation of tertiary sewage treatment (Oviatt et al. 2017; Oczkowski et al. 2018). Also, waters have warmed by 1.4–1.6°C since 1960 (Fulweiler et al. 2015), as well as the offshore waters on the North Atlantic continental shelf by 0.37°C (Chen et al. 2020). This combination of altered nutrient status and background of elevated temperature may contribute to conditions augmenting *Pseudo‐nitzschia* HABs in Narragansett Bay.

The contrasting dynamics we observed between *Pseudo‐nitzschia* originating from offshore vs. resident populations have implications for HAB formation in Narragansett Bay and regionally, as these data show the importance of species‐specific dynamics in toxic events. Although no closures occurred from September 2017 to November 2019, we established baseline data for seasonal *Pseudo‐nitzschia* spp. assemblages that regularly recur, especially during detectable particulate domoic acid maxima in the fall and summer. The presence of particularly toxic species, such as *P. australis*, during periods with elevated domoic acid motivates future studies aimed at linking particulate domoic acid with species abundance (using methods such as quantitative PCR). This will inform when environmental and species intersections are concerning in regard to HAB formation.

## Conclusion

In this study, we showed a high‐resolution time series of particulate domoic acid concentrations and *Pseudo‐nitzschia* spp. assemblages which revealed seasonal patterns in Narragansett Bay that appear to recur annually. These observations will assist in predicting timing of future domoic acid closures, with the identification of possible abiotic factors, temperature and nitrogen, contributing to toxin production. High‐throughput sequencing of the ITS1 rDNA region has provided important multi‐species composition data, including identifying rare but potentially highly toxic species (i.e., *P. australis*) and previously unknown taxa to the region along with the composition of resident assemblages that may be driven to higher toxin production. Because the highest particulate domoic acid concentrations were observed at the Narragansett Bay mouths, these entrances may serve as sites for sentinel mussels and other tools to monitor the introduction of offshore species. Narragansett Bay managers and shellfish harvesters must prepare for possible HAB event increases, as this study has shown low levels of particulate domoic acid present in the fall and summer that reoccur each year and their magnitude may change as it did in 2017 leading to a domoic acid closure. Additional temporal introduction of highly toxic species such as *P. australis*, responding to shifts in oceanographic patterns and warming water temperatures, may provide increasingly favorable conditions for more severe HABs in Narragansett Bay and along the Northeast Atlantic.

## Conflict of Interest

None declared.

## Supporting information


**Appendix S1** Supporting information

## Data Availability

The raw sequencing reads are available on NCBI's Short Read Archive (Bioproject PRJNA690940). All ASVs identified in this study as *Pseudo‐nitzschia* were deposited into NCBI GenBank under accession numbers MW447658‐MW447770. All *Pseudo‐nitzschia* ASVs, assigned taxonomy, and presence or absence in samples used in this study are publicly available through the NSF Biology & Chemical Oceanography Data Management Office (BCO‐DMO) (Jenkins and Bertin 2021*a*). Domoic acid data, *Pseudo‐nitzschia* spp. cell counts, and environmental metadata are also available through NSF BCO‐DMO (Jenkins and Bertin 2021*b*).

## References

[lno12189-bib-0001] Amato, A. , W. H. C. F. Kooistra , J. H. Levialdi Ghiron , D. G. Mann , T. Pröschold , and M. Montresor . 2007. Reproductive isolation among sympatric cryptic species in marine diatoms. Protist 158: 193–207. doi:10.1016/j.protis.2006.10.001 17145201

[lno12189-bib-0002] Anderson, C. R. , and others . 2019. Scaling up from regional case studies to a global harmful algal bloom observing system. Front. Mar. Sci. 6: 250. doi:10.3389/fmars.2019.00250

[lno12189-bib-0003] Auro, M. E. , and W. P. Cochlan . 2013. Nitrogen utilization and toxin production by two diatoms of the *Pseudo‐nitzschia pseudodelicatissima* complex: *P. cuspidata* and *P. fryxelliana* . J. Phycol. 49: 156–169. doi:10.1111/jpy.12033 27008397

[lno12189-bib-0004] Bates, S. S. , and others . 1989. Pennate diatom *Nitzschia pungens* as the primary source of domoic acid, a toxin in shellfish from eastern Prince Edward Island, Canada. Can. J. Fish. Aquat. Sci. 46: 1203–1215. doi:10.1139/f89-156

[lno12189-bib-0005] Bates, S. S. , D. J. Douglas , G. J. Doucette , and C. Léger . 1995. Enhancement of domoic acid production by reintroducing bacteria to axenic cultures of the diatom *Pseudo‐nitzschia multiseries* . Nat. Toxins 3: 428–435. doi:10.1002/nt.2620030605 8612005

[lno12189-bib-0006] Bates, S. S. , C. Léger , and K. M. Smith . 1996. Domoic acid production by the diatom *Pseudo‐nitzschia multiseries* as a function of division rate in silicate‐limited chemostat culture. Intergovernmental Oceanographic Commission of UNESCO. 163–166.

[lno12189-bib-0007] Bates, S. S. , K. A. Hubbard , N. Lundholm , M. Montresor , and C. P. Leaw . 2018. *Pseudo‐nitzschia*, *Nitzschia*, and domoic acid: New research since 2011. Harmful Algae 79: 3–43. doi:10.1016/j.hal.2018.06.001 30420013

[lno12189-bib-0008] Bolyen, E. , and others . 2019. Reproducible, interactive, scalable and extensible microbiome data science using QIIME 2. Nat. Biotechnol. 37: 852–857. doi:10.1038/s41587-019-0209-9 31341288 PMC7015180

[lno12189-bib-0009] Brunson, J. K. , and others . 2018. Biosynthesis of the neurotoxin domoic acid in a bloom‐forming diatom. Science 361: 1356–1358. doi:10.1126/science.aau0382.Biosynthesis 30262498 PMC6276376

[lno12189-bib-0010] Callahan, B. J. , P. J. McMurdie , M. J. Rosen , A. W. Han , A. J. A. Johnson , and S. P. Holmes . 2016. DADA2: High resolution sample inference from Illumina amplicon data. Nat. Methods 13: 581–583. doi:10.1038/nmeth.3869 27214047 PMC4927377

[lno12189-bib-0011] Canesi, K. L. , and T. A. Rynearson . 2016. Temporal variation of *Skeletonema* community composition from a long‐term time series in Narragansett Bay identified using high‐throughput DNA sequencing. Mar. Ecol. Prog. Ser. 556: 1–16. doi:10.3354/meps11843

[lno12189-bib-0012] Casteleyn, G. , and others . 2008. *Pseudo‐nitzschia pungens* (Bacillariophyceae): a cosmopolitan diatom species? Harmful Algae 7: 241–257. doi:10.1016/j.hal.2007.08.004

[lno12189-bib-0013] Chappell, P. D. , E. V. Armbrust , K. A. Barbeau , R. M. Bundy , J. W. Moffett , J. Vedamati , and B. D. Jenkins . 2019. Patterns of diatom diversity correlate with dissolved trace metal concentrations and longitudinal position in the Northeast Pacific coastal‐offshore transition zone. Mar. Ecol. Prog. Ser. 609: 69–86. doi:10.3354/meps12810

[lno12189-bib-0014] Chen, Z. , Y. Kwon , K. Chen , P. Fratantoni , G. Gawarkiewicz , and T. M. Joyce . 2020. Long‐term SST variability on the Northwest Atlantic continental shelf and slope. Geophys. Res. Lett. 47: e2019GL085455. doi:10.1029/2019GL085455

[lno12189-bib-0015] Clark, S. , K. A. Hubbard , D. M. Anderson , D. J. McGillicuddy Jr. , D. K. Ralston , and D. W. Townsend . 2019. *Pseudo‐nitzschia* bloom dynamics in the Gulf of Maine: 2012‐2016. Harmful Algae 88: 101656. doi:10.1016/j.hal.2019.101656 31582158 PMC6779423

[lno12189-bib-0016] Clark, S. , K. A. Hubbard , D. J. McGillicuddy Jr. , D. K. Ralston , and S. Shankar . 2021. Investigating *Pseudo‐nitzschia australis* introduction to the Gulf of Maine with observations and models. Cont. Shelf Res. 228: 104493. doi:10.1016/j.csr.2021.104493 36213213 PMC9536250

[lno12189-bib-0017] Clarke, K. R. , and M. Ainsworth . 1993. A method of linking multivariate community structure to environmental variables. Mar. Ecol. Prog. Ser. 92: 205–219. doi:10.3354/meps092205

[lno12189-bib-0018] Delegrange, A. , A. Lefebvre , F. Gohin , L. Courcot , and D. Vincent . 2018. *Pseudo‐nitzschia* sp. diversity and seasonality in the southern North Sea, domoic acid levels and associated phytoplankton communities. Estuar. Coast. Shelf Sci. 214: 194–206. doi:10.1016/j.ecss.2018.09.030

[lno12189-bib-0019] Dixon, P. 2003. VEGAN, a package of R functions for community ecology. J. Veg. Sci. 14: 927–930. doi:10.1111/j.1654-1103.2003.tb02228.x

[lno12189-bib-0020] Dortch, Q. , and others . 1997. Abundance and vertical flux of *Pseudo‐nitzschia* in the northern Gulf of Mexico. Mar. Ecol. Prog. Ser. 146: 249–264. doi:10.3354/meps146249

[lno12189-bib-0021] Falkowski, P. G. 1994. The role of phytoplankton photosynthesis in global biogeochemical cycles. Photosynth. Res. 39: 235–258. doi:10.1007/BF00014586 24311124

[lno12189-bib-0022] Fox, J. , and S. Weisberg . 2019. An R companion to applied regression. SAGE Publications.

[lno12189-bib-0023] Fulweiler, R. W. , A. J. Oczkowski , K. M. Miller , C. A. Oviatt , and M. E. Q. Pilson . 2015. Whole truths vs. half truths—and a search for clarity in long‐term water temperature records. Estuar. Coast. Shelf Sci. 157: A1–A6. doi:10.1016/j.ecss.2015.01.021

[lno12189-bib-0024] Gallacher, S. , G. Howard , P. Hess , E. MacDonald , M. Kelly , M. Mackenzie , and P. Gillibrand . 2001. The occurrence of amnesic shellfish poisons in shellfish from Scottish waters, p. 30–33. *In* W. Turrell [ed.], Harmful algal blooms 2000: Proceedings of the ninth international conference on harmful algal blooms. UNESCO.

[lno12189-bib-0025] Garnier, S. 2018. viridis: Default Color Maps from “matplotlib”.

[lno12189-bib-0026] Gonçalves Neto, A. , J. A. Langan , and J. B. Palter . 2021. Changes in the Gulf stream preceded rapid warming of the Northwest Atlantic shelf. Commun. Earth Environ. 2: 74. doi:10.1038/s43247-021-00143-5

[lno12189-bib-0085] Grattan, L. , Boushey, C., Liang, Y., Lefebvre, K., Castellon, L., Roberts, K., Toben, A., & Morris, J. 2018. Repeated Dietary Exposure to Low Levels of Domoic Acid and Problems with Everyday Memory: Research to Public Health Outreach. Toxins, 10(3), 103. 10.3390/toxins10030103 29495583 PMC5869391

[lno12189-bib-0027] Hagström, J. A. , E. Granéli , M. O. P. Moreira , and C. Odebrecht . 2011. Domoic acid production and elemental composition of two *Pseudo‐nitzschia multiseries* strains, from the NW and SW Atlantic Ocean, growing in phosphorus‐ or nitrogen‐limited chemostat cultures. J. Plankton Res. 33: 297–308. doi:10.1093/plankt/fbq102

[lno12189-bib-0028] Hargraves, P. E. , and L. Maranda . 2002. Potentially toxic or harmful microalgae from the northeast coast. Northeast. Nat. 9: 81–120. doi:10.2307/3858576

[lno12189-bib-0029] Hasle, G. R. 2002. Are most of the domoic acid‐producing species of the diatom genus *Pseudo‐nitzschia* cosmopolites? Harmful Algae 1: 137–146. doi:10.1016/S1568-9883(02)00014-8

[lno12189-bib-0030] Hoagland, P. , D. M. Anderson , Y. Kaoru , and A. W. White . 2002. The economic effects of harmful algal blooms in the United States: Estimates, assessment issues, and information needs. Estuaries 25: 819–837. doi:10.1007/BF02804908

[lno12189-bib-0031] Howard, M. D. A. , W. P. Cochlan , N. Ladizinsky , and R. M. Kudela . 2007. Nitrogenous preference of toxigenic *Pseudo‐nitzschia australis* (Bacillariophyceae) from field and laboratory experiments. Harmful Algae 6: 206–217. doi:10.1016/j.hal.2006.06.003

[lno12189-bib-0032] Hubbard, K. A. , G. Rocap , and E. V. Armbrust . 2008. Inter‐ and intraspecific community structure within the diatom genus *Pseudo‐nitzschia* (Bacillariophyceae). J. Phycol. 44: 637–649. doi:10.1111/j.1529-8817.2008.00518.x PMC3873754 24376283

[lno12189-bib-0033] Hubbard, K. A. , C. E. Olson , and E. V. Armbrust . 2014. Molecular characterization of *Pseudo‐nitzschia* community structure and species ecology in a hydrographically complex estuarine system (Puget Sound, Washington, USA). Mar. Ecol. Prog. Ser. 507: 39–55. doi:10.3354/meps10820 27239082 PMC4882114

[lno12189-bib-0034] Jaccard, P. 1912. The distribution of the flora in the alpine zone. New Phytol. 11: 37–50. doi:10.1111/j.1469-8137.1912.tb05611.x

[lno12189-bib-0035] Jellet, J. , V. Trainer , S. Bates , and I. Somerset . 2006. Characteristics and applications of the Jellett rapid tests. Proceedings of the Fifth International Conference on Molluscan Shellfish Safety. 6–12.

[lno12189-bib-0036] Jenkins, B. D. , and M. Bertin . 2021 *a*. Presence or absence of amplicon sequence variants (ASVs) recovered from samples which are described in DATASET 01, *Pseudo‐nitzschia* spp. from weekly samples and offshore cruises with the Northeast U.S. Shelf (NES) Long‐Term Ecological Research (LTER). Biological and Chemical Oceanography Data Management Office Data Sets. 10.26008/1912/bco-dmo.847495.1

[lno12189-bib-0037] Jenkins, B. D. , and M. Bertin . 2021 *b*. *Pseudo‐nitzschia* spp. cell counts, nutrients water temperature and salinity, and concentrations of the toxin domoic acid from weekly samples and offshore cruises with the Northeast U.S. Shelf (NES) Long‐Term Ecological Research (LTER). Biological and Chemical Oceanography Data Management Office Data Sets. 10.26008/1912/bco-dmo.847448.1

[lno12189-bib-0038] Kaczmarska, I. , C. Reid , J. L. Martin , and M. B. J. Moniz . 2008. Morphological, biological, and molecular characteristics of the diatom *Pseudo‐nitzschia delicatissima* from the Canadian Maritimes. Botany 86: 763–772. doi:10.1139/B08-046

[lno12189-bib-0039] Karentz, D. , and T. Smayda . 1984. Temperature and seasonal occurrence patterns of 30 dominant phytoplankton species in Narragansett Bay over a 22‐year period (1959‐1980). Mar. Ecol. Prog. Ser. 18: 277–293. doi:10.3354/meps018277

[lno12189-bib-0040] Kassambara, A. , and F. Mundt . 2020. factoextra: Extract and visualize the results of multivariate data analyses.

[lno12189-bib-0041] Katoh, K. , K. Misawa , K. Kuma , and T. Miyata . 2002. MAFFT: A novel method for rapid multiple sequence alignment based on fast Fourier transform. Nucleic Acids Res. 30: 3059–3066. doi:10.1093/nar/gkf436 12136088 PMC135756

[lno12189-bib-0042] Katoh, K. , and D. M. Standley . 2013. MAFFT multiple sequence alignment software version 7: Improvements in performance and usability. Mol. Biol. Evol. 30: 772–780. doi:10.1093/molbev/mst010 23329690 PMC3603318

[lno12189-bib-0043] Kudela, R. M. , J. Q. Lane , and W. P. Cochlan . 2008. The potential role of anthropogenically derived nitrogen in the growth of harmful algae in California, USA. Harmful Algae 8: 103–110. doi:10.1016/j.hal.2008.08.019

[lno12189-bib-0044] Lefebvre, K. A. , and others . 1999. Detection of domoic acid in northern anchovies and California Sea lions associated with an unusual mortality event. Nat. Toxins 7: 85–92. doi:10.1002/(sici)1522-7189(199905/06)7:3<85::aid-nt39>3.0.co;2-q 10647509

[lno12189-bib-0045] Lelong, A. , H. Hégaret , P. Soudant , and S. S. Bates . 2012. *Pseudo‐nitzschia* (Bacillariophyceae) species, domoic acid and amnesic shellfish poisoning: Revisiting previous paradigms. Phycologia 51: 168–216. doi:10.2216/11-37.1

[lno12189-bib-0046] Levitan, O. , and others . 2015. Remodeling of intermediate metabolism in the diatom *Phaeodactylum tricornutum* under nitrogen stress. Proc. Natl. Acad. Sci. USA 112: 412–417. doi:10.1073/pnas.1419818112 25548193 PMC4299248

[lno12189-bib-0047] Lundholm, N. , Ø. Moestrup , G. R. Hasle , and K. Hoef‐Emden . 2003. A study of the *Pseudo‐nitzschia pseudodelicatissima*/*cuspidata* complex (Bacillariophyceae): What is *P. pseudodelicatissima*? J. Phycol. 39: 797–813. doi:10.1046/j.1529-8817.2003.02031.x

[lno12189-bib-0048] Lundholm, N. , Ø. Moestrup , Y. Kotaki , K. Hoef‐Emden , C. Scholin , and P. Miller . 2006. Inter‐ and intraspecific variation of the *Pseudo‐nitzschia delicatissima* complex (Bacillariophyceae) illustrated by rRNA probes, morphological data and phylogenetic analyses. J. Phycol. 42: 464–481. doi:10.1111/j.1529-8817.2006.00211.x

[lno12189-bib-0049] Markina, Z. V. , and N. A. Aizdaicher . 2016. The effect of lowered salinity of sea water on the growth and photosynthetic pigment content in three strains of the microalgae *Pseudo‐nitzschia pungens* (Grunow ex. P.T. Cleve) Hasle, 1993 (Bacillariophyta). Russ. J. Mar. Biol. 42: 414–418. doi:10.1134/S1063074016050060

[lno12189-bib-0050] Martin, M. 2011. Cutadapt removes adapter sequences from high‐throughput sequencing reads. EMBnet.journal 17: 10–12. doi:10.14806/ej.17.1.200

[lno12189-bib-0051] McCabe, R. , and others . 2016. An unprecedented coastwide toxic algal bloom linked to anomalous ocean conditions. Geophys. Res. Lett. 43: 366–376. doi:10.1002/2016GL070023 PMC5129552 27917011

[lno12189-bib-0052] McKibben, S. M. , W. Peterson , A. M. Wood , V. L. Trainer , M. Hunter , and A. E. White . 2017. Climatic regulation of the neurotoxin domoic acid. Proc. Natl. Acad. Sci. USA 114: 239–244. doi:10.1073/pnas.1606798114 28069959 PMC5240689

[lno12189-bib-0053] McMurdie, P. J. , and S. Holmes . 2013. Phyloseq: An R package for reproducible interactive analysis and graphics of microbiome census data. PLoS One 8: e61217. doi:10.1371/journal.pone.0061217 23630581 PMC3632530

[lno12189-bib-0054] Míguez, A. , and L. Fernlindez . 1996. First detection of domoic acid in Galicia (NW of Spain), p. 143–145. *In* T. Yasumoto , Y. Oshima , and Y. Fukuyo [eds.], Harmful and toxic algal blooms. Intergovernmental Oceanographic Commission of UNESCO.

[lno12189-bib-0055] Miller, R. L. , and D. L. Kamykou . 1986. Effects of temperature, salinity, irradiance and diurnal periodicity on growth and photosynthesis in the diatom *Nitzschia americana*: Light‐saturated growth. J. Phycol. 22: 339–348. doi:10.1111/j.1529-8817.1986.tb00033.x

[lno12189-bib-0056] National Shellfish Sanitation Program (NSSP) . 2019. Guide for the control of molluscan shellfish: 2019 revision.

[lno12189-bib-0057] Oczkowski, A. , and others . 2018. How the distribution of anthropogenic nitrogen has changed in Narragansett Bay (RI, USA) following major reductions in nutrient loads. Estuaries Coasts 41: 2260–2276. doi:10.1007/s12237-018-0435-2 30971866 PMC6452444

[lno12189-bib-0058] Orive, E. , L. Pérez‐Aicua , H. David , K. García‐Etxebarria , A. Laza‐Martínez , S. Seoane , and I. Miguel . 2013. The genus *Pseudo‐nitzschia* (Bacillariophyceae) in a temperate estuary with description of two new species: *Pseudo‐nitzschia plurisecta* sp. nov. and *Pseudo‐nitzschia abrensis* sp. nov. J. Phycol. 49: 1192–1206. doi:10.1111/jpy.12130 27007637

[lno12189-bib-0059] Orsini, L. , G. Procaccini , D. Sarno , and M. Montresor . 2004. Multiple rDNA ITS‐types within the diatom *Pseudo‐nitzschia delicatissima* (Bacillariophyceae) and their relative abundances across a spring bloom in the Gulf of Naples. Mar. Ecol. Prog. Ser. 271: 87–98. doi:10.3354/meps271087

[lno12189-bib-0060] Oviatt, C. A. 2004. The changing ecology of temperate coastal waters during a warming trend. Estuaries 27: 895–904. doi:10.1007/BF02803416

[lno12189-bib-0061] Oviatt, C. , L. Smith , J. Krumholz , C. Coupland , H. Stoffel , A. Keller , M. C. McManus , and L. Reed . 2017. Managed nutrient reduction impacts on nutrient concentrations, water clarity, primary production, and hypoxia in a north temperate estuary. Estuar. Coast. Shelf Sci. 199: 25–34. doi:10.1016/j.ecss.2017.09.026

[lno12189-bib-0062] Pan, Y. , D. V. Subba Rao , and K. H. Mann . 1996. Changes in domoic acid production and cellular chemical composition of the toxigenic diatom *Pseudo‐nitzschia multiseries* under phosphate limitation. J. Phycol. 32: 371–381. doi:10.1111/j.0022-3646.1996.00371.x

[lno12189-bib-0063] Pedregosa, F. , and others . 2011. Scikit‐learn: Machine learning in python. J. Mach. Learn. Res. 12: 2825–2830.

[lno12189-bib-0064] Quijano‐Scheggia, S. I. , E. Garcés , N. Lundholm , Ø. Moestrup , K. Andree , and J. Camp . 2009. Morphology, physiology, molecular phylogeny and sexual compatibility of the cryptic *Pseudo‐nitzschia delicatissima* complex (Bacillariophyta), including the description of *P. arenysensis* sp. nov. Phycologia 48: 492–509. doi:10.2216/08-21.1

[lno12189-bib-0065] R Core Team . 2020. R: A language and environment for statistical computing.

[lno12189-bib-0066] RI Harmful Algal Bloom (HAB) and Shellfish Biotoxin Monitoring and Contingency Plan . 2020. http://www.dem.ri.gov/programs/benviron/water/shellfsh/pdf/habplan.pdf

[lno12189-bib-0067] RStudio . 2016. RStudio: Integrated development for R.

[lno12189-bib-0068] Ryan, J. P. , and others . 2017. Causality of an extreme harmful algal bloom in Monterey Bay, California, during the 2014–2016 Northeast Pacific warm anomaly. Geophys. Res. Lett. 44: 5571–5579. doi:10.1002/2017GL072637

[lno12189-bib-0069] Rynearson, T. A. , S. A. Flickinger , and D. N. Fontaine . 2020. Metabarcoding reveals temporal patterns of community composition and realized thermal niches of *Thalassiosira* spp. (Bacillariophyceae) from the Narragansett Bay Long‐Term Plankton Time Series. Biology (Basel) 9: 19. doi:10.3390/biology9010019 31963344 PMC7168904

[lno12189-bib-0070] Sahraoui, I. , S. S. Bates , D. Bouchouicha , H. Hadj Mabrouk , and A. Sakka Hlaili . 2011. Toxicity of *Pseudo‐nitzschia* populations from Bizerte lagoon, Tunisia, Southwest Mediterranean, and first report of domoic acid production by *P. brasiliana* . Diatom Res. 26: 293–303. doi:10.1080/0269249X.2011.597990

[lno12189-bib-0071] Schlitzer, R. 2002. Interactive analysis and visualization of geoscience data with ocean data view. Comput. Geosci. 28: 1211–1218. doi:10.1016/S0098-3004(02)00040-7

[lno12189-bib-0072] Sison‐Mangus, M. P. , S. Jiang , R. M. Kudela , and S. Mehic . 2016. Phytoplankton‐associated bacterial community composition and succession during toxic diatom bloom and non‐bloom events. Front. Microbiol. 7: 1433. doi:10.3389/fmicb.2016.01433 27672385 PMC5018474

[lno12189-bib-0073] Smayda, T. J. , and The Bunker C Community . 1959–1997. Narragansett Bay Plankton Time Series. Graduate School of Oceanography, University of Rhode Island. Available from: NABATS.org.

[lno12189-bib-0074] Tammilehto, A. , T. Gissel Nielsen , B. Krock , E. Friis Møller , and N. Lundholm . 2015. Induction of domoic acid production in the toxic diatom *Pseudo‐nitzschia seriata* by calanoid copepods. Aquat. Toxicol. 159: 52–61. doi:10.1016/j.aquatox.2014.11.026 25521565

[lno12189-bib-0075] Tatters, A. O. , F. Fu , and D. A. Hutchins . 2012. High CO_2_ and silicate limitation synergistically increase the toxicity of *Pseudo‐nitzschia fraudulenta* . PLoS One 7: e32116. doi:10.1371/journal.pone.0032116 22363805 PMC3283721

[lno12189-bib-0076] Thessen, A. E. , H. A. Bowers , and D. K. Stoecker . 2009. Intra‐ and interspecies differences in growth and toxicity of *Pseudo‐nitzschia* while using different nitrogen sources. Harmful Algae 8: 792–810. doi:10.1016/j.hal.2009.01.003

[lno12189-bib-0077] Tol, P. 2018. Colour Schemes. SRON/EPS/TN/09‐002. SRON/EPS/TN/09‐002, Prospects for detecting eV‐scale sterile neutrinos from a galactic supernova.

[lno12189-bib-0078] Trainer, V. L. , S. S. Bates , N. Lundholm , A. E. Thessen , W. P. Cochlan , N. G. Adams , and C. G. Trick . 2012. *Pseudo‐nitzschia* physiological ecology, phylogeny, toxicity, monitoring and impacts on ecosystem health. Harmful Algae 14: 271–300. doi:10.1016/j.hal.2011.10.025

[lno12189-bib-0079] Turner . 2019. Trilogy Laboratory fluorometer user's manual. Turner Designs.

[lno12189-bib-0080] White, T. , T. Bruns , S. Lee , and J. Taylor . 1990. Amplification and direct sequencing of fungal ribosomal RNA genes for phylogenetics, p. 315–322. *In* PCR Protocols: A guide to methods and applications. Academic Press, Inc.

[lno12189-bib-0081] Wickham, H. 2016. ggplot2: Elegant graphics for data analysis. Springer‐Verlag.

[lno12189-bib-0082] Work, T. M. , B. Barr , A. M. Beale , L. Fritz , M. A. Quilliam , and J. L. C. Wright . 1993. Epidemiology of domoic acid poisoning in brown pelicans (*Pelecanus occidentalis*) and Brandt's cormorants (*Phalacrocorax penicillatus*) in California. J. Zoo Wildl. Med. 24: 54–62.

[lno12189-bib-0083] Zabaglo, K. , E. Chrapusta , B. Bober , A. Kaminski , M. Adamski , and J. Bialczyk . 2016. Environmental roles and biological activity of domoic acid: A review. Algal Res. 13: 94–101. doi:10.1016/j.algal.2015.11.020

[lno12189-bib-0084] Zimmermann, J. , R. Jahn , and B. Gemeinholzer . 2011. Barcoding diatoms: Evaluation of the V4 subregion on the 18S rRNA gene, including new primers and protocols. Org. Divers. Evol. 11: 173–192. doi:10.1007/s13127-011-0050-6

